# Small local variations in B-form DNA lead to a large variety of global geometries which can accommodate most DNA-binding protein motifs

**DOI:** 10.1186/1472-6807-9-24

**Published:** 2009-04-24

**Authors:** Arvind Marathe, Deepti Karandur, Manju Bansal

**Affiliations:** 1 Molecular Biophysics Unit, Indian Institute of Science, Bangalore - 12, India

## Abstract

**Background:**

An important question of biological relevance is the polymorphism of the double-helical DNA structure in its free form, and the changes that it undergoes upon protein-binding. We have analysed a database of free DNA crystal structures to assess the inherent variability of the free DNA structure and have compared it with a database of protein-bound DNA crystal structures to ascertain the protein-induced variations.

**Results:**

Most of the dinucleotide steps in free DNA display high flexibility, assuming different conformations in a sequence-dependent fashion. With the exception of the AA/TT and GA/TC steps, which are 'A-phobic', and the GG/CC step, which is 'A-philic', the dinucleotide steps show no preference for A or B forms of DNA. Protein-bound DNA adopts the B-conformation most often. However, in certain cases, protein-binding causes the DNA backbone to take up energetically unfavourable conformations. At the gross structural level, several protein-bound DNA duplexes are observed to assume a curved conformation in the absence of any large distortions, indicating that a series of normal structural parameters at the dinucleotide and trinucleotide level, similar to the ones in free B-DNA, can give rise to curvature at the overall level.

**Conclusion:**

The results illustrate that the free DNA molecule, even in the crystalline state, samples a large amount of conformational space, encompassing both the A and the B-forms, in the absence of any large ligands. A-form as well as some non-A, non-B, distorted geometries are observed for a small number of dinucleotide steps in DNA structures bound to the proteins belonging to a few specific families. However, for most of the bound DNA structures, across a wide variety of protein families, the average step parameters for various dinucleotide sequences as well as backbone torsion angles are observed to be quite close to the free 'B-like' DNA oligomer values, highlighting the flexibility and biological significance of this structural form.

## Background

Watson and Crick proposed the double-helical structure for DNA in 1953, and almost simultaneously two forms were postulated on the basis of fibre diffraction analysis – the B-form DNA corresponded to the Watson and Crick structure and was found to occur in conditions of high humidity and low salt concentration, while the A-form occurred in conditions of lower humidity and higher salt concentration. Gross structural features for these as well as other polymorphic forms of DNA were refined during the next 25 years, using fibre diffraction data [1,2]. The two forms of DNA were mainly characterised in terms of features such as sugar pucker [3-5], glycosyl torsion angle [6], base pair orientation and the groove widths [7], apart from the helical parameters rise and twist. However, it was only in the 1980s that the atomic details of the two forms were characterised. The first crystal structure of a B-form DNA was solved in 1981 [8], and was found to have significant sequence-dependant variability, with an average roll per dinucleotide step of 0.5 ± 5.2°, an average local helical twist of 35.6 ± 4.4° and an average slide of 0.2 ± 0.5 Å. Subsequent analyses of other crystal structures confirmed the sequence dependent effects observed here [9-12]. A-DNA, which was first crystallised by McCall [13], was found to have an average roll of 6.8 ± 2.6°, an average local helical twist of 30.8 ± 1.2° and an average slide of -1.5 ± 0.4 Å [10,12,14]. However, since the overall features of the two crystal structures were close to the fibre models of B and A forms, it was assumed that the two forms correspond to two stable minima that the DNA could assume and transition from one form to another would involve some energetic costs. As crystallographic methods improved and the number and variety of x-ray crystal structures of DNA increased, this idea began to lose ground. While most oligonucleotide structures solved during 1980–2000 had roll and twist values that ranged from exclusively A-like to exclusively B-like, a few appeared to show features intermediate between A-DNA and B-DNA, to a mixture of both types. [15-18]. Thus it appeared that A and B-form DNA were not well separated stable minima, and the dinucleotide steps in oligomeric DNA could assume conformations that ranged from B-like to intermediate to A-like [2]. In addition, several other forms of synthetic DNA were also solved, which did not fit the canonical A-like or B-like conformation [2]. Against this wide ranging polymorphism of the double-helical DNA molecule, particularly at the dinucleotide step level, the RNA duplex crystal structures, that were solved around the same time [19-24], stood out for their rigidity, and their conformational proximity to the A-RNA fibre model, independent of the sequence. In this study, we have analysed a large dataset of free RNA oligomers to verify the conformational rigidity of Watson-Crick basepaired RNA duplexes and then used it as a template against which to measure the A-like characteristics of each dinucleotide step as well as overall structure of both free and protein-bound DNA.

Several studies in the late 1990s also suggested that not only the classical B-form of DNA but also the A-form had biological relevance. Based on a comparison of free B-DNA oligomers and protein-bound DNA, it was suggested [25,26] that protein binding causes DNA to assume A-like or an A-B intermediate conformation in terms of roll and twist. Subsequently it was shown that a new parameter, Z_*p*_, could be used to discriminate between A-like or B-like dinucleotide steps more reliably than roll or twist, and that entire structures could be classified as A-like or B-like in terms of their Z_*p *_values, irrespective of the local variations in their roll and twist values [14,27]. Lu et al [14] highlighted the fact that in DNA structures bound to a few prominent protein families, the protein-bound region was induced to take up an A-like conformation as defined by Z_*p*_. However, the above mentioned studies [25,26], that compared free and bound DNA, considered the overall B-form of the free oligomers as a reference, and not the inherent 'A-philicity' [28-32] of dinucleotide steps in the bound region. Given that atleast in a few cases, the putative binding region is known to assume an A-like conformation in its free form [33,34], inclusion of A-DNA oligomer structures also in the analysis might provide better insights into the intrinsic preferences of a DNA sequence and help distinguish these from protein induced structural effects. Only one study compares the free and protein-bound forms of DNA, taking into consideration the A-form of DNA [35]. Several other studies have implicated the variations in roll, especially at pyrimidine-purine steps, to be responsible for DNA bending and curvature [25,36-41], in ways critical for the binding of the protein.

While the DNA dinucleotide steps were under scrutiny for their role in specifically binding to a protein, the DNA backbone was also shown to be involved in more than 50% of all the contacts between amino acids and the DNA in regulatory protein-DNA complexes [42]. Hence several studies have also focused on how the variations in the DNA backbone might act as an indirect readout signal for protein recognition and binding [43-49]. In DNA oligomers, the sugar phosphate backbone was believed to be rigid, compared to the variation in local step geometry, defined by two neighbouring basepairs. The sugar ring assumed C_3_'-endo conformation in A-DNA [3] and C_2_'-endo in B-DNA [9]. The related backbone torsion angle *δ *was found to assume values of about 84° for A-DNA [3] and about 128° for B-DNA [4,5]. The torsion angles *ε *and *ζ *were observed to assume two conformations-BI and BII in B-DNA [9] but only the BI conformation in A-DNA [9]. *α *and *γ *were found to show anticorrelated variation in A-form duplexes [3,24,50,51], but were generally found to take up the *g*^-^, *g*^+ ^conformation in B-DNA [9]. However, recent studies have shown that unlike oligomers, the backbone in a significant proportion of nucleotides in bound DNA assumes non-classical conformations [52]. There have also been attempts to analyse the backbone torsion angles, taking into account the correlation between more than two torsion angles and group them into seven distinct states [53,54]. In this study, we have adapted this methodology [53] and analysed the variation in backbone parameters with respect to variation in dinucleotide step parameters across different datasets.

A crucial question of biological relevance is how the variations in DNA structure at the basepair, base-step and backbone level contribute to the overall structure of the molecule, and its implications for protein binding. A related question is how changes caused by protein binding at the local structural level affect the overall DNA structure. There have been efforts to go beyond dinucleotide steps and analyse the properties of all possible tetranucleotide, hexanucleotide and octanucleotide fragments using molecular dynamics simulation studies [53,55,56]. However, most of the high resolution DNA double helical crystal structures, especially those of free DNA, are too short in length, to allow a meaningful statistical analysis of all possible trinucleotide or higher order steps. The other approach is to try and quantify the overall DNA structure, in terms of parameters such as DNA curvature, bendability or stability. The importance of DNA curvature was first realized when it was observed that even unbound genomic DNA could have a well-defined, inherent curvature [57,58]. Since most of the curved DNA observed in the early days were observed to have stretches of adenines, the initial models of DNA curvature, such as the 'wedge model' [59] and the 'junction model' [60], traced the origin of curvature to the presence of A-tracts, in phase with the DNA helical repeat. However, these models had to be abandoned when it was shown that sequences lacking in AA dinucleotides also adopted a curved structure [61]. Thus new models which took into account variation in the geometries of all ten dinucleotide steps were proposed [62-64]. However, owing to difficulties in tracing a uniform path for the DNA axis in three dimensions, there is no standard methodology for calculating DNA curvature, despite its obvious importance in biological functions. Various measures of quantifying DNA curvature such as the radius of a circle fitted to the basepair centres projected onto a plane [63,65], the ratio of the end-to-end distance of the DNA molecule to the actual path traced by the DNA axis [63,65-69], ratio of the moments of inertia of an ellipsoid fitted to the molecule [63,65-71] as well as the angles between two local helix axes vectors corresponding to two successive dinucleotide steps have been proposed and implemented [64,69,72]. However, each of these methods has its advantages and limitations, and no single method can unambiguously quantify all possible curved conformations adopted by DNA molecules. Hence a combination of all or several of these methods along with a close inspection of the local level distortions is required to fully understand the curvature of any given structure.

In this study, we have analysed an exhaustive dataset of protein-DNA complexes, and compared it with a complete, high resolution dataset of free DNA oligomers, without pre-classifying them as A-DNA or B-DNA. We have also separately analysed a dataset of DNA bound by proteins via a Helix-Turn-Helix (HTH) motif. The HTH motif is not only the most well-characterised, but also the most commonly occurring DNA-binding motif, and is present in a wide range of transcription factors. The HTH motif consists of two alpha helices linked by a turn region that protrudes out of the surface of the protein [73-75]. The second helix, usually referred to as the 'recognition helix', fits into the major groove of the DNA, and is involved in direct or indirect interactions with the DNA [74-76]. While the HTH motif has been studied extensively, the structural features of the DNA to which it binds have not been analysed in detail. The present analysis provides some interesting insights into the conformational flexibility of the DNA molecule, and reveals that many of the conformations observed in bound DNA, both at the local dinucleotide step level, and the gross structural level, are also accessible to unbound DNA, while a few conformations are solely induced by protein binding.

## Results

The structural parameters of three datasets of DNA – free oligomers, protein-bound DNA (excluding HTH motif-bound DNA) and HTH motif-bound DNA, and one dataset of RNA oligomers were analysed in order to gain a complete perspective of the features of DNA both within each set and also across the sets. As RNA is known to assume only A-like conformation, the RNA dataset was used as a reference point for A-like conformation and also to characterise the basepair effects from those due to the ribose sugar ring in RNA. The RNA dataset was observed to be rich in steps containing the G:C basepair, and had remarkably low percentage of steps containing only the A:U basepair (table 1). The free DNA dataset consists of a large proportion of the steps GG (23.9%) and CG (18.0%) (table 1). A significant number of these steps were found to occur in structures which were classified by the Nucleic Acid Database [77] as "A-DNA". A large number of these steps are indeed found to have high Z_*p *_values in the present analysis, matching our criteria for an A-like dinucleotide step, as defined in the next section. The free dataset also contains 5 structures with the Drew-Dickerson sequence d(CGCGAATTCGCG). These and other A-tract containing sequences primarily contribute to the high occurrence of AA steps (11.1%) in the free dataset. The HTH dataset consists of DNA bound by a wide variety of proteins ranging across 22 SCOP [78] classes, and includes 3 ternary TATA Binding Protein-Transcription Factor IIB-TATA-box (hereafter referred to as TBP-TFIIB-TA-DNA) complexes and 6 Catabolite Activator Protein-DNA (hereafter referred to as CAP-DNA) complexes (additional file 1). In the TBP-TFIIB-TA-box DNA ternary complexes, the HTH motif is present in the transcription factor TFIIB, which binds to the DNA immediately upstream of the TATA-box region. The complex dataset contains 8 TATA Binding Protein-DNA (hereafter referred to as TBP-DNA) complexes, which lack TFIIB, and hence have been excluded from the HTH dataset (additional file 1). Interestingly, the protein-bound datasets also have a significant proportion of CA/TG steps, which have been implicated in the kinks observed in several structures [79].

**Table 1:** Occurrence of the ten unique dinucleotide steps in the four datasets (numbers in parentheses indicate percentage occurrence).

	Occurence							
	
	RNA	Free			Complex		HTH	
Dinucleotide Sequence*		All	A-like (Z_*p *_> 1.3 Å)	B-like (Z_*p *_≤ 0.8 Å)	All	Excl. TEH†	All	Excl. TC ‡
AA/TT	15 (5.4)	45 (11.1)	NA	45 (21.5)	211 (17.2)	126 (16.2)	259 (16.6)	208 (15.0)

AG/CT	29 (10.5)	17 (4.2)	3 (1.5)	14 (6.7)	151 (12.3)	92 (11.8)	138 (8.9)	120 (8.7)

GA/TC	19 (6.9)	42 (10.3)	2 (1.0)	40 (19.1)	142 (11.6)	71 (9.1)	164 (10.5)	151 (10.9)

GG/CC	36 (13.0)	97 (23.9)	90 (45.9)	7 (3.3)	120 (9.8)	84 (10.8)	134 (8.6)	118 (8.5)

AC/GT	36 (13.0)	23 (5.7)	13 (6.6)	10 (4.8)	134 (10.9)	98 (12.6)	188 (12.1)	173 (12.5)

AT/AT	9 (3.3)	28 (6.9)	4 (2.0)	24 (11.5)	102 (8.3)	54 (6.9)	155 (9.9)	143 (10.3)

GC/GC	44 (15.9)	39 (9.6)	25 (12.8)	13 (6.2)	63 (5.1)	48 (6.2)	83 (5.3)	71 (5.1)

CA/TG (BI)	43 (15.6)	17 (4.2)	13 (6.6)	4 (1.9)	93 (7.6)	71 (9.1)	152 (9.7)	139 (10.0)

CA/TG (BII)	NA	12 (3.0)	NA	12 (5.7)	60 (4.9)	57 (7.3)	63 (4.0)	60 (4.3)

CG/CG	37 (13.4)	73 (18.0)	43 (21.9)	30 (14.4)	50 (4.1)	32 (4.1)	82 (5.3)	75 (5.4)

TA/TA	8 (3.0)	13 (3.2)	3 (1.5)	10 (4.8)	101 (8.2)	45 (5.8)	141 (9.0)	126 (9.1)

TOTAL	276	406	196	209	1227	778	1559	1384

†TEH – TBP, Endonuclease and Hyperthermophile chromosomal protein SAC7D containing structures (excluded)

‡TC – TBP and CAP containing structures (excluded)

* RNA oligomers contain Uracil (U) instead of Thymine (T). d(U)-containing steps are not included for the DNA datasets. The CA/TG step was classified as having BII conformation, if either the Cytosine or the Thymine base or both were observed to have the backbone torsion angle values *ε *- *ζ *> 0.

### Variations of the dinucleotide step parameters

**Figure 1:**
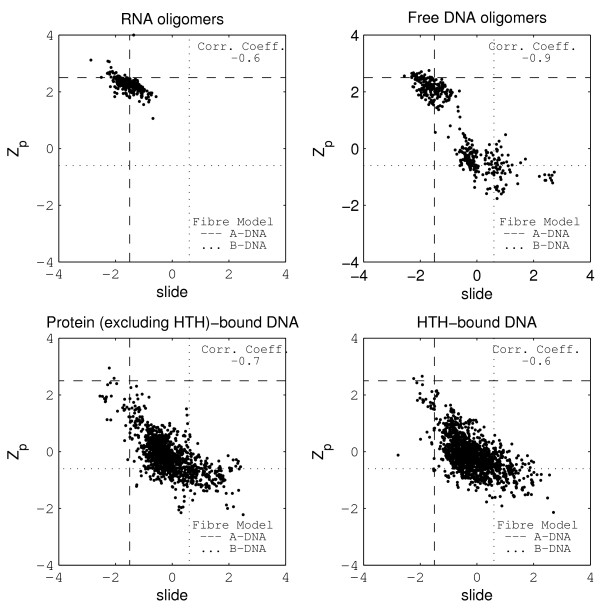
**Z_*p *_versus Slide for the RNA, free DNA oligomers, protein-bound DNA (not containing the HTH motif) and HTH-bound DNA datasets**. Dashed and dotted lines have been drawn to highlight the values for the A and B-DNA fibre models.

**Figure 2:**
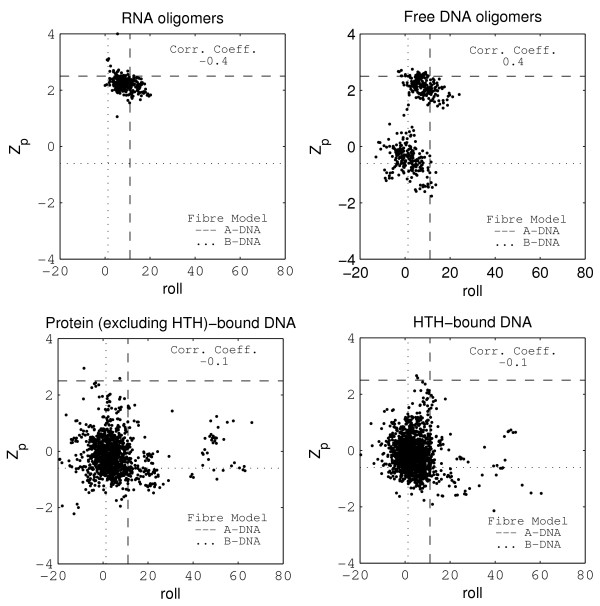
**Z_*p *_versus Roll for the RNA, free DNA oligomers, protein-bound DNA (not containing the HTH motif) and HTH-bound DNA datasets**. Dashed and dotted lines have been drawn to highlight the values for the A and B-DNA fibre models.

Among the six dinucleotide step parameters that measure the relative rotational and translational motions between adjacent basepairs about the x, y and z-axis (see 'Methods'), tilt, shift and rise were observed to have very little variation within and across the three DNA datasets, and so have not been reported here. On the other hand, in conformity with earlier studies [25-27], the parameters roll, twist and slide, as well as the parameter Z_*p *_(described in the 'Methods' section), were found to be excellent indicators for analysing the sequence dependent conformational flexibility of a DNA molecule. To highlight the characteristic features of each dinucleotide step in free as well as bound DNA, the dinucleotide step parameters Z_*p *_and slide are listed in tables 2 and 3, while figure 1 shows the variation of Z_*p *_versus slide. The corresponding values for roll and twist are listed in tables 4 and 5, while the variation of Z_*p *_versus roll is shown in figure 2.

**Table 2:** Mean and standard deviation values (given in parentheses, reported only for datasets with size ≥ 5) for Z_*p *_for the ten dinucleotide steps containing Watson-Crick basepairs.

	Z_*p*_						
	RNA	Free		Complex		HTH	
Dinucleotide Sequence*		A-like (Z_*p *_> 1.3 Å)	B-like (Z_*p *_≤ 0.8 Å)	All	Excl. TEH†	All	Excl. TC ‡

AA/TT	2.1 (0.2)	NA	-0.5 (0.4)	-0.5 (0.5)	-0.4 (0.4)	-0.4 (0.5)	-0.4 (0.4)

AG/CT	2.2 (0.3)	2.2 NA	-0.6 (0.6)	0.0 (0.8)	-0.3 (0.6)	0.0 (0.6)	-0.0 (0.6)

GA/TC	2.4 (0.4)	2.1 NA	-0.2 (0.4)	-0.1 (0.7)	-0.3 (0.5)	-0.2 (0.5)	-0.1 (0.5)

GG/CC	2.4 (0.2)	2.2 (0.3)	-0.8 (0.4)	0.1 (0.7)	-0.1 (0.6)	0.4 (0.9)	0.3 (0.9)

AC/GT	2.1 (0.3)	2.1 (0.3)	-0.4 (0.2)	-0.0 (0.5)	-0.1 (0.4)	0.0 (0.6)	0.1 (0.6)

AT/AT	2.0 (0.2)	2.3 NA	-0.3 (0.5)	-0.0 (0.6)	-0.1 (0.4)	-0.1 (0.5)	-0.1 (0.5)

GC/GC	2.2 (0.2)	2.0 (0.3)	-0.3 (0.5)	-0.3 (0.6)	-0.3 (0.5)	-0.0 (0.5)	0.0 (0.5)

CA/TG (BI)	2.2 (0.4)	2.0 (0.3)	-0.4 NA	-0.0 (0.8)	-0.2 (0.5)	-0.1 (0.5)	-0.1 (0.5)

CA/TG (BII)	NA	NA	-0.9 (0.3)	-0.4 (0.5)	-0.4 (0.5)	-0.4 (0.5)	-0.4 (0.5)

CG/CG	2.4 (0.3)	2.1 (0.3)	-0.5 (0.5)	-0.0 (0.8)	0.1 (0.6)	-0.1 (0.6)	-0.1 (0.6)

TA/TA	2.1 (0.2)	1.9 NA	-0.6 (0.7)	-0.2 (0.6)	-0.2 (0.5)	-0.2 (0.6)	-0.2 (0.5)

Overall	2.2 (0.3)	2.1 (0.3)	-0.5 (0.5)	-0.1 (0.7)	-0.2 (0.5)	-0.1 (0.6)	-0.1 (0.6)

Fibre-Model		2.5	-0.6				

†TEH – TBP, Endonuclease and Hyperthermophile chromosomal protein SAC7D containing structures (excluded)

‡TC – TBP and CAP containing structures (excluded)

The other specifications are as detailed in the caption to table 1.

**Table 3:** Mean and standard deviation values (given in parentheses, reported only for datasets with size ≥ 5) for slide for the ten dinucleotide steps containing Watson-Crick basepairs.

	Slide						
	RNA	Free		Complex		HTH	
Dinucleotide Sequence*		A-like (Z_*p *_> 1.3 Å)	B-like (Z_*p *_≤ 0.8 Å)	All	Excl. TEH†	All	Excl. TC ‡

AA/TT	-1.4 (0.4)	NA	-0.1 (0.4)	0.0 (0.7)	-0.2 (0.4)	-0.1 (0.5)	-0.2 (0.4)

AG/CT	-1.5 (0.3)	-1.4 NA	0.1 (0.5)	-0.2 (0.7)	-0.1 (0.7)	-0.2 (0.5)	-0.2 (0.5)

GA/TC	-1.6 (0.3)	-1.9 NA	0.0 (0.5)	-0.1 (0.7)	0.1 (0.6)	-0.1 (0.6)	-0.2 (0.5)

GG/CC	-1.8 (0.3)	-1.7 (0.3)	0.3 (0.4)	-0.3 (0.7)	-0.1 (0.7)	-0.5 (0.7)	-0.4 (0.7)

AC/GT	-1.3 (0.4)	-1.2 (0.4)	0.0 (0.6)	-0.6 (0.4)	-0.5 (0.2)	-0.5 (0.5)	-0.5 (0.4)

AT/AT	-1.2 (0.2)	-1.6 NA	-0.3 (0.3)	-0.5 (0.4)	-0.5 (0.3)	-0.6 (0.3)	-0.6 (0.3)

GC/GC	-1.3 (0.3)	-1.1 (0.3)	0.4 (0.5)	-0.1 (0.6)	-0.0 (0.7)	-0.3 (0.5)	-0.3 (0.5)

CA/TG (BI)	-1.6 (0.3)	-1.4 (0.2)	0.1 NA	-0.1 (0.9)	-0.0 (0.7)	-0.1 (0.7)	-0.2 (0.7)

CA/TG (BII)	NA	NA	2.2 (0.7)	0.9 (0.9)	0.9 (1.0)	0.7 (0.8)	0.6 (0.8)

CG/CG	-1.9 (0.3)	-1.8 (0.3)	0.7 (0.5)	0.1 (0.9)	-0.0 (0.6)	0.4 (0.7)	0.4 (0.7)

TA/TA	-1.6 (0.3)	-1.4 NA	0.5 (0.6)	0.0 (1.0)	-0.2 (0.9)	-0.0 (1.0)	-0.1 (0.9)

Overall	-1.5 (0.4)	-1.6 (0.4)	0.2 (0.8)	-0.1 (0.8)	-0.1 (0.7)	-0.2 (0.7)	-0.2 (0.6)

Fibre-Model		-1.5	0.6				

†TEH – TBP, Endonuclease and Hyperthermophile chromosomal protein SAC7D containing structures (excluded)

‡TC – TBP and CAP containing structures (excluded)

The other specifications are as detailed in the caption to table 1.

**Table 4:** Mean and standard deviation values (given in parentheses, reported only for datasets with size ≥ 5) for roll for the ten dinucleotide steps containing Watson-Crick basepairs.

	Roll						
	RNA	Free		Complex		HTH	
Dinucleotide Sequence*		A-like (Z_*p *_> 1.3 Å)	B-like (Z_*p *_≤ 0.8 Å)	All	Excl. TEH†	All	Excl. TC‡

AA/TT	9.3 (2.9)	NA	0.2 (4.0)	6.6 (13.0)	1.7 (7.6)	2.8 (7.8)	1.5 (4.1)

AG/CT	8.6 (2.2)	3.2 NA	3.5 (4.7)	4.3 (7.4)	3.3 (5.2)	5.4 (8.6)	4.3 (4.9)

GA/TC	9.3 (4.9)	11.6 NA	0.6 (3.7)	1.3 (7.2)	3.0 (4.4)	2.6 (4.8)	2.5 (4.7)

GG/CC	8.3 (2.4)	7.5 (4.1)	6.1 (2.7)	4.7 (4.5)	5.7 (4.1)	5.3 (4.1)	5.5 (4.2)

AC/GT	6.3 (3.3)	3.9 (3.9)	0.2 (4.9)	2.0 (5.0)	2.9 (3.4)	2.5 (4.1)	2.4 (4.0)

AT/AT	11.3 (3.6)	1.3 NA	-0.4 (3.8)	4.6 (11.7)	0.5 (4.5)	1.5 (5.8)	0.7 (3.5)

GC/GC	6.4 (2.9)	6.6 (4.2)	-3.7 (5.7)	1.1 (4.7)	1.1 (5.1)	2.9 (4.2)	2.7 (4.1)

CA/TG (BI)	11.9 (3.9)	10.1 (4.8)	6.1 NA	6.9 (5.7)	8.0 (4.0)	8.2 (7.8)	7.3 (3.9)

CA/TG (BII)	NA	NA	-4.8 (4.9)	-0.4 (7.4)	-0.5 (7.6)	2.8 (9.3)	2.6 (8.4)

CG/CG	10.8 (4.5)	11.9 (4.3)	6.1 (5.0)	9.0 (14.0)	7.5 (3.3)	6.5 (8.7)	6.3 (8.1)

TA/TA	12.1 (5.0)	12.0 NA	2.4 (6.1)	14.3 (19.1)	3.9 (6.8)	5.6 (10.6)	3.5 (6.4)

Overall	9.0 (4.0)	8.2 (4.8)	1.2 (5.3)	5.0 (10.7)	3.3 (5.9)	4.0 (7.4)	3.4 (5.3)

Fibre-Model		11.1	1.3				

†TEH – TBP, Endonuclease and Hyperthermophile chromosomal protein SAC7D containing structures (excluded)

‡TC – TBP and CAP containing structures (excluded)

The other specifications are as detailed in the caption to table 1.

**Table 5:** Mean and standard deviation values (given in parentheses, reported only for datasets with size ≥ 5) for twist for the ten dinucleotide steps containing Watson-Crick basepairs.

	Twist						
	RNA	Free		Complex		HTH	
Dinucleotide Sequence*		A-like (Z_*p *_> 1.3 Å)	B-like (Z_*p *_≤ 0.8 Å)	All	Excl. TEH†	All	Excl. TC ‡

AA/TT	31.1 (3.3)	NA	36.1 (3.9)	30.8 (7.3)	34.4 (3.8)	33.9 (6.0)	35.1 (3.5)

AG/CT	31.0 (3.3)	31.8 NA	32.5 (7.0)	33.4 (5.1)	34.1 (4.5)	33.0 (5.6)	33.5 (4.0)

GA/TC	31.5 (2.6)	24.1 NA	37.7 (3.4)	33.9 (5.0)	35.0 (3.6)	34.8 (4.3)	34.9 (4.4)

GG/CC	30.6 (2.2)	30.5 (3.2)	31.2 (5.3)	33.4 (4.0)	33.5 (4.2)	32.3 (4.4)	32.4 (4.5)

AC/GT	31.0 (2.7)	33.1 (1.4)	33.6 (5.7)	30.9 (3.7)	31.2 (3.2)	30.7 (4.1)	30.8 (4.0)

AT/AT	31.1 (1.7)	33.6 NA	33.5 (3.9)	28.4 (6.5)	32.3 (3.6)	31.5 (4.0)	32.0 (2.8)

GC/GC	32.4 (5.6)	33.0 (2.7)	38.2 (2.8)	32.6 (5.7)	33.2 (5.7)	32.5 (4.7)	32.5 (4.9)

CA/TG (BI)	31.2 (2.9)	29.1 (2.6)	36.5 NA	33.9 (4.9)	33.5 (3.7)	33.0 (5.0)	33.2 (4.3)

CA/TG (BII)	NA	NA	49.1 (2.4)	39.3 (5.9)	39.5 (6.0)	38.0 (5.2)	38.2 (4.5)

CG/CG	29.7 (2.1)	29.3 (3.7)	32.2 (5.6)	36.0 (6.4)	37.2 (3.0)	35.0 (5.8)	35.1 (5.5)

TA/TA	30.0 (1.1)	27.7 NA	34.7 (6.4)	29.3 (9.1)	35.1 (7.3)	34.9 (7.6)	36.4 (6.0)

Overall	31.1 (3.4)	30.6 (3.5)	35.8 (5.9)	32.4 (6.4)	34.1 (4.8)	33.3 (5.5)	33.8 (4.7)

Fibre-Model		30.8	36.0				

†TEH – TBP, Endonuclease and Hyperthermophile chromosomal protein SAC7D containing structures (excluded)

‡TC – TBP and CAP containing structures (excluded)

The other specifications are as detailed in the caption to table 1.

The RNA oligomer dataset assumes mean values of high positive Z_*p *_(2.2 ± 0.3 Å), negative slide (-1.5 ± 0.4 Å), high roll (9.0 ± 4.0°) and low twist (31.1 ± 3.4°), all close to the values observed for the fibre models of A-form DNA helices [80]. The low values of the standard deviations for all four parameters for individual steps, as well as for the entire dataset, confirms the conformational rigidity of the RNA structures. The sugar-phosphate backbone torsion angles *χ *and *δ *and the phase angle P were also observed to assume A-DNA fibre model-like conformation, for the entire dataset. Even steps that have previously been reported to be A-phobic in DNA are observed to be entirely A-like in RNA. This confirms the observation that the presence of even a single ribose sugar causes the entire structure to assume A-like conformation [24], while the presence of uracil in place of thymine also facilitates the A-form structure, particularly for AA/UU, GA/UC and AG/CU type of steps. Thus, the RNA dataset, with its well-defined and rigid boundaries stands in sharp contrast to the free and protein-bound DNA datasets and its parameters can be used as a criteria to define A-like conformation in DNA.

For the free DNA dataset, as seen in figure 1, a distinct bimodal distribution is observed for Z_*p *_and slide. The two distinct clusters for this dataset arise primarily because Z_*p *_assumes two distinctly different values with a clear separation between them. Using the RNA dataset as a template, we assigned as A-like, those DNA steps that lie within three standard deviations of the mean Z_*p *_value for the RNA dataset viz. Z_*p *_> 1.3 Å. The boundary for B-like conformation was assigned at 0.8 Å, based on visual inspection of figure 1. The points with Z_*p *_between 0.8 and 1.3 Å were considered to have intermediate conformation. With the exception of one datapoint, all free DNA oligomer steps can be classified as A-like or B-like in terms of Z_*p*_. The A-like cluster has a mean of 2.1 Å, close to the value for RNA oligomers and the other cluster has a mean value of -0.5 Å, which corresponds to the fibre model value for B-DNA (-0.6 Å) [14]. For all the four datasets, slide was observed to correlate well with Z_*p *_for the overall data (figure 1), as well as for individual dinucleotide sequences. In contrast, roll does not show a significant correlation with Z_*p *_(figure 2), nor do the roll and twist parameters (additional file 2, figure 1) show any bimodal character.

#### A-philicity of dinucleotides in DNA structures

Efforts have been made several decades back to characterise individual dinucleotide steps as being A-phobic (or B-philic) (AA, CA and GA) or A-philic (GG, AG and AC) [28-31] on the basis of their ability to induce a B to A transition in solution. A more recent study on a larger dataset [32] reclassified the GA step as neutral and the AG step as B-philic. Our analysis confirms that the AA and GG steps are highly B-philic and A-philic respectively, in terms of their preference for Z_*p *_and slide values. The GA and AG steps in our dataset also show high preference for B-like conformation, except for very few steps which have A-like parameters. AC, which has earlier been reported to be A-philic, as well as AT, GC, TA and CG steps were observed to display both A-like or B-like values, though AT and TA show a preference for B-form.

CA/TG steps assume both A and B types of conformations in terms of Z_*p *_and slide, the steps with B-like values occurring in structures with A-tracts that are overall B-like, and steps with A-like values occurring in structures with a large number of C:G basepair containing steps that have high, or A-like, Z_*p *_values. B-like CA steps themselves assume two types of conformations in terms of roll, twist and the backbone torsion angles *ε *and *ζ*, namely BI and BII, thus confirming the highly flexible nature of this dinucleotide step, with no marked preference for A or B like geometries.

Thus it appears that in free DNA oligomers, the overall structure assumes A-like or B-like conformation depending on its sequence, particularly the proportion of AA/TT and GG/CC steps. Only AA, and to a lesser extent GA steps show strong preference for B-form, while GG is truly A-philic. All the other dinucleotide steps do not appear to have a strong intrinsic preference for A-like or B-like conformation, but assume a particular conformation depending on the conformation of neighbouring steps, as suggested by recent solution studies [32].

In the protein-bound DNA datasets, most of the structures were found to exclusively have B-like values for Z_*p*_, if the above-mentioned criteria for A-like and B-like DNA is used. Unlike the free dataset, no structure from the complex or HTH dataset was observed to have entirely A-like conformation. Even for an A-philic step such as GG, for which 92.8% of the steps in the free dataset take up an A-like geometry, about 90.0% of the datapoints in the complex dataset and about 71.6% of the datapoints in the HTH dataset were observed to have B-like values of Z_*p*_, with only 5.0% and 12.7% of datapoints respectively, showing an A-like value for Z_*p*_. Only a few steps in the DNA-binding region of some structures were observed to have A-like or near A-like characteristics. These complexes belong to a few specific families, such as the polymerases, endonucleases and transposases, and the structural features of these duplexes have been described in the 'Discussion' section.

#### Roll and twist are not good discriminators of A-form versus B-form

Roll and twist span a very wide range of values for the three DNA datasets, as evident from their values listed in tables 4 and 5. Unlike Z_*p *_and slide, there is no clear bimodal distribution for roll and twist for the free dataset, with the values varying in a continuous negatively correlated fashion, from high negative roll and very large twist to positive roll and low twist (additional file 2, figure 1). In the free DNA dataset, steps which have been classified as A-like or B-like based on their Z_*p *_values, have been listed separately in tables 4 and 5.

As mentioned above, CA steps show three types of conformations-one in which Z_*p*_, slide, roll and twist have typical A-like values and two different conformations, wherein Z_*p *_is B-like. CA steps with B-like Z_*p *_values are observed to assume either normal slide and twist with positive roll or high positive slide, large twist and negative roll. This bimodal distribution of the B-like CA steps has been observed in several previous studies [10,65,81,82]. These steps also show a correlated variation in the backbone torsion angles, *ε *and *ζ *in both strands, with the low twist and positive roll steps having *ε *and *ζ *in the *t*, *g*^- ^(or BI) conformation, while the large twist and negative roll steps have *ε *and *ζ *in the *g*^-^, *t *(or BII) conformation [9,83]. When a CA step in the BII conformation occurs adjacent to an AG step such that it forms a CAG triplet, the AG step is often observed to have a high roll and a very low twist. This feature is observed in several DNA structures irrespective of whether the steps have bound ions [84,85] or are present free [16]. These CA steps and the adjacent AG steps do not show any correlated variation in Z_*p *_and slide, which have B-like values, with these CA steps being characterised by large positive slide values. The AG steps occurring adjacent to other steps do not assume this conformation. The high roll and low twist values of these AG steps, which are B-like in terms of Z_*p*_, skew the averages for roll (3.5 ± 4.7°) and twist (32.5 ± 7.0°) to A-like values.

In addition to the CA step, 8 of the 13 'B-like' GC steps are also observed to assume the BII conformation for one or both of the guanine backbone torsion angles, and have a corresponding negative value of roll and a large value of twist. As a result, 'B-like' GC steps have a negative average value for roll (-3.7 ± 5.7°) and a large average value for twist (38.2 ± 2.8°). AA steps, which are exclusively B-like in terms of Z_*p*_, have mean roll and twist values of 0.2 ± 4.0 and 36.1 ± 3.9 respectively, indicating that these steps are B-like in terms of roll and twist also. Among the other dinucleotide steps, the GG and CG steps have A-like mean values for both roll and twist, irrespective of whether their Z_*p *_value is A-like or B-like. For the remaining steps, mean values for roll and twist follow the trend set by Z_*p*_. However, the large values of standard deviations for all the steps, including B-philic steps such as AA and GA, and an A-philic step such as GG, indicate that a significant number of steps have intermediate conformation in terms of roll and twist. This is also illustrated by the Z_*p *_versus roll plot in figure 2, which does not show any clear demarcation between the A and B like steps.

The large, continuous variation in roll and twist has been observed earlier [35] and is also evident in the twist versus roll plot for the bound-DNA datasets (additional file 2, figure 1), where a large number of the mean roll and twist values are intermediate between those assumed by the A and B-DNA fibre models (tables 4, 5). The higher standard deviations for all the parameters in most of the steps in the bound datasets, when compared to the free DNA dataset, prompted us to individually examine the structures that are responsible for the high standard deviations. For the complex dataset, nearly all the datapoints with more than 3*σ *deviation from the mean roll or twist values of the free B-like DNA oligomer dataset were found to occur in structures belonging to three families-the TBP-bound DNA, the endonuclease-bound DNA and the hyperthermophile SAC7D protein-bound DNA. DNA bound to the integration host factor also undergoes significant distortions in roll and twist. For the HTH dataset, nearly all the datapoints with more than 3*σ *deviation from the mean roll or twist values of the free B-like DNA oligomer dataset are contributed by the TATA-box-TFIIB and CAP-bound DNA structures. On excluding these structures, the mean values are much closer to B-DNA fibre model values, with low standard deviations, and comparable to those obtained for B-like steps in the free dataset. Significantly, the exclusion of the above mentioned structural families made no significant difference in the mean values of Z_*p *_and slide for any of the steps (tables 2 and 3), indicating that the B-like DNA structure can accommodate large variations in roll and twist parameters, with no corresponding change in Z_*p*_and slide. This is further corroborated by the low correlation between either roll or twist with Z_*p *_or slide, for all the steps across the three DNA datasets. The low correlation between Z_*p*_and roll is clearly evident in figure 2.

Interestingly, several CAG triplets in the nucleosome structures [86] show the same unusual combination of parameters observed for the CAG triplets in some oligomers, with the CA step in BII conformation while the AG step has high roll and low twist values so that the overall roll and twist values for the two steps are similar to that in canonical B-DNA.

### Free DNA oligomers can be classified as A-DNA or B-DNA in terms of Z_*p*_

At the overall structural level, most of the DNA duplexes in the free dataset can be entirely classified as A-like or B-like in terms of Z_*p*_, with the exception of 5 structures, 196D, 1P4Z, 1ZFA, 399D and 441D, wherein one or two of the steps show Z_*p *_values which differ significantly from that seen for the overall structure. Even the crystal structure of the G:C rich sequence d(CATGGGCCCATG) (1DC0), reported as an A ↔ B intermediate [87], assumes A-like Z_*p *_and slide values for all the steps, and hence can be described as A-like, though the roll and twist values show considerable variation.

The global x-displacement, helical rise, inclination and helical twist as well as the major and minor groove widths, described in the 'Methods' section, are also considered to be indicators of the overall A-like or B-like nature of a DNA structure, and we compared the average values of these parameters across the datasets (additional file 2, table 1). Since entire structures in the free dataset could be assigned as A-DNA or B-DNA on the basis of Z_*p*_, the averages of the global x-displacement, helical rise, inclination and helical twist for all the non-terminal basepairs within all the A-DNA structures were classified as 'A-DNA' values for the respective parameters. Similar procedure was adopted for the basepair orientation parameters within all the B-DNA structures to obtain 'B-DNA' values. As expected, the RNA dataset assumes A-like values for all the parameters, while the values for the A-like and B-like free DNA datasets being very close to their corresponding fibre model values reaffirms that the overall free DNA oligomer structures can be classified as A-like or B-like.

For both the bound DNA datasets, while the global helical rise is observed to be strongly B-DNA like, with very little variation, the global x-displacement, inclination and helical twist take up values between those for the 'A-DNA' and 'B-DNA' datasets, but closer to B-DNA. The groove width values for the bound DNA datasets for both the major and minor grooves are 'B-DNA' like. The rather large values of standard deviation for inclination and helical twist in case of the free 'B-DNA' dataset implies that B-DNA, in its free form, might be able to access the conformations observed in bound DNA.

### Variations of the DNA backbone

**Figure 3:**
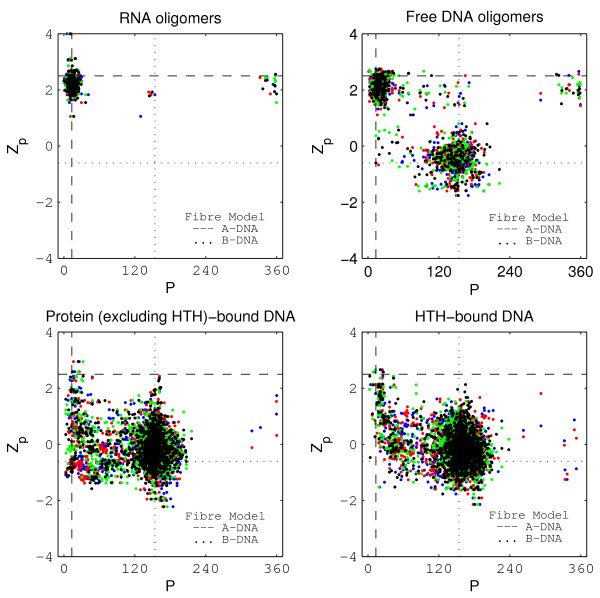
**Z_*p *_versus sugar pseudorotation phase angle P for the four datasets**. The four nucleotides constituting a basepaired dinucleotide step corresponding to a particular Z_*p *_value have been colour coded as follows: *5'-end Strand 1 – Blue; 3'-end Strand 1 – Red; 5'-end Strand 2 – Green; 3'-end Strand 2 – Black*.

The backbone torsion angle *δ*, defined by C_5_'-C_4_'-C_3_'-O_3_', the pseudorotation phase angle P [1], which characterises the sugar ring pucker, and the glycosidic torsion angle *χ*, defined by O_4_'-C_1_'-N_1_-C_2 _in pyrimidines and O_4_'-C_1_'-N_9_-C_4 _in purines, have the most characteristically distinct values in A and B-DNA [4,5]. Figure 3 shows the variation of Z_*p *_with respect to the angle P. The two torsion angles *χ *(additional file 2, figure 2) and *δ *(additional file 2, figure 3) show similar behaviour. Note that each dinucleotide step described by a single Z_*p *_value encompasses four values of sugar pucker and glycosidic torsions, corresponding to the 4 bases constituting a dinucleotide step. As expected, the entire RNA dataset shows A-like conformation. Free DNA shows two clusters that correspond to A-like and B-like regions described by previous studies [4,5]. An inspection of the four values of *χ*, *δ *and P that constitute each step in the free DNA dataset reveals that for a step with A-like Z_*p*_, all four values for all three angles were A-like and for a B-like step, all four values were B-like. A few exceptions were also observed in a few structures, where in a single step with A-like Z_*p *_value, one of the four P angles was observed to be B-like (1ZEY, 1ZF6, 1ZF87, 1ZFA) and vice versa (1EHV, 1DUO, 1ENN, 1IKK, 1SK5, 1ZFA, 307D, 423D, 463D, 477D, 7BNA) (see additional file 1 for detailed references corresponding to all the PDB id's). The B-like nature of backbone parameters also holds true with respect to B-like steps in the bound datasets, where all four values for all the three angles are usually B-like. Exceptions occur in the structures that displayed unusual behaviour in the local step parameters, and these have been described in detail in the relevant section.

**Figure 4:**
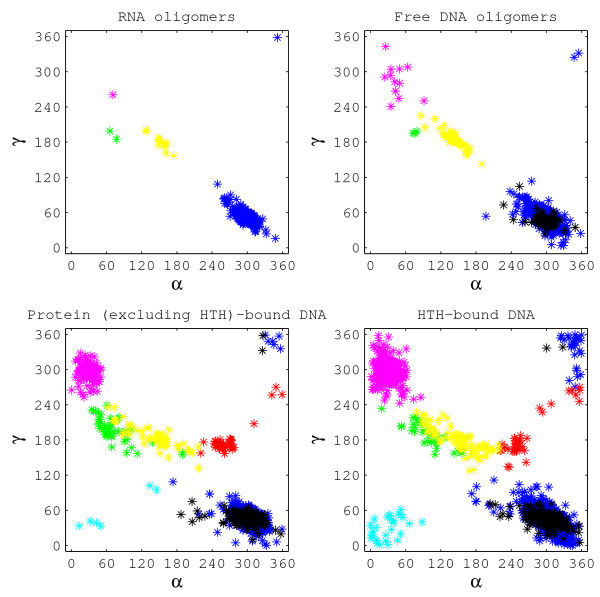
**The correlation between the backbone torsion angles *γ *vs *α *for all four datasets is shown in different colours, indicating the seven states as defined in **[53]. The seven states are colour coded as follows: *State 1 – Blue; State 2 – Red; State 3 – Green; State 4 – Cyan; State 5 – Yellow; State 6 – Magenta; State 7 – Black*.

We have also analysed the conformationally flexible torsion angles *α*, *γ*, *ε*-*ζ*, using a modified version of the algorithm of Dixit et al [53] such that it applied to torsion angles across a step. Table 6 and figure 4 show the distribution of the seven states described by this algorithm, across all dinucleotide steps. The RNA dataset displays classical behaviour, with an overwhelming majority of the steps assuming canonical values for *α*, *γ*, *ε*-*ζ*, viz. *g*^-^, *g*^+^, BI (state 1). For the three DNA datasets, there is much greater conformational flexibility, with *α*, *γ*, *ε*-*ζ *= *g*^-^, *g*^+^, BII (state 7), being the predominant non-canonical conformation. However, there is a significantly lower occurrence of the state 7 conformation in the bound datasets. Protein binding seems to induce a few B-like steps to assume the *α*, *γ *= *t*, *t *(state 3 or state 5) conformation, that is not preferred by free B-form DNA.

**Table 6:** Occurence of the 7 states, defined according to [53], across the four datasets (Numbers in parenthesis indicate percentage occurrence.)

	DESCRIPTION				Occurrence			
STATE	*α *(°)	*γ *(°)	*ε*-*ζ *(°)	RNA	Free		Complex	HTH
					A-like (Z_*p *_> 1.3 Å)	B-like (Z_*p *_≤ 0.8 Å)		
1	150–360	0–125 or 270–360	BI	534 (96.7)	335 (85.5)	339 (81.1)	1933 (78.8)	2259 (72.5)
2	220–360	125–270	-	0 (0.0)	0 (0.0)	0 (0.0)	38 (1.5)	40 (1.3)
3	0–220	125–240	BII	2 (0.4)	0 (0.0)	3 (0.7)	48 (2.0)	34 (1.1)
4	0–150	0–125	-	0 (0.0)	0 (0.0)	0 (0.0)	6 (0.2)	29 (0.9)
5	0–220	125–240	BI	15 (2.7)	47 (12.0)	1 (0.2)	58 (2.4)	97 (3.1)
6	0–220	240–270	-	1 (0.2)	8 (2.0)	4 (1.0)	125 (5.1)	366 (11.7)
7	150–360	0–125 or 270–360	BII	0 (0.0)	2 (0.5)	71 (17.0)	246 (10.0)	293 (9.4)

The most noteworthy difference between the free and the bound datasets was observed in the case of state 6, where the allowed ranges for *α *and *γ *occur between 0–220° and 240–270° respectively [53]. However, all the datapoints belonging to state 6, in our four datasets, occupy a much narrower range, close to the *α*, *γ *= *g*^+^, *g*^- ^conformation, that has been reported to be energetically unfavourable [52]. While its presence is negligible in the free dataset, a substantial proportion of the steps in the HTH dataset assume this conformation (table 6). A significant proportion (22.4%) of the steps in the HTH dataset that assumed this conformation were observed to be AT, with one or both of the thymine bases in these steps taking up the unusual *α *and *γ *values. Although no restriction was placed on the *ε*-*ζ *value for state 6, almost all the datapoints for this state were observed to have the BI conformation. The steps with state 6 conformation occurred with equal frequency in the bound as well as the unbound regions of the DNA, and were not observed to assume unusual values for any other structural parameter.

### Variations at the trinucleotide level

**Figure 5:**
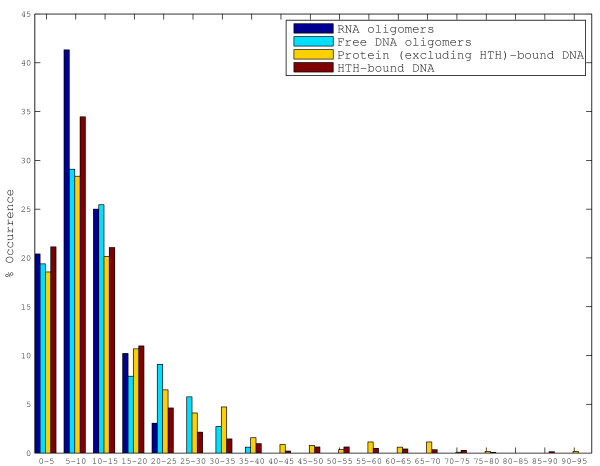
**Histogram showing the percentage occurrence for successive bending angle values (in °) for the four datasets (values for terminal triplets have been excluded)**. RNA oligomers generally have small bending angles while the protein-bound DNA shows a large range of bending angles.

The absence of large protein-induced DNA distortion is also apparent when one examines the successive bending angles (figure 5). The successive bending angle is directly proportional to the difference in successive roll values, and can be considered to be a measure of the local bending at the trinucleotide level. The RNA dataset generally shows small successive bending angle values (with 96.9% of the values < 20°), as would be expected from a dataset with nearly uniform roll values. Of the protein-bound datasets, the HTH dataset shows surprising results. 55.7% of the triplets in the HTH dataset have bending angles between 0–10° when compared to 48.5% in the free dataset, indicating that a majority of the HTH-bound triplets tend to be less distorted than even the free triplets. 46.9% of the triplets in the complex dataset occur in this range. The trend is reversed for the range between 20–30°, which could be considered to indicate moderately 'distorted' triplets, with 14.8% of the free triplets occurring in this range when compared to only 6.8% of the HTH triplets and 10.6% of the triplets in the complex dataset. However, as noted before, binding by proteins belonging to a few specific families appears to cause large distortions in roll and twist values of a few dinucleotide steps in both the bound datasets. For example, bending angles for the steps that are distorted by TBP and CAP, in both the protein-bound datasets, range from 50° to 80°. An inspection of the stretches of DNA in the regions with high bending angles in free DNA oligomers revealed that dinucleotides with very high magnitude of roll and very large or very small twist are almost completely absent, yet a series of successive near normal roll and twist values frequently give rise to reasonably high bending angles at the triplet level. Protein binding, and especially HTH binding, does not appear to distort the DNA anymore than when it is in the free state, except in the case of a few special families.

### Protein-induced distortions in DNA structure

#### Dinucleotide step level

Most of the significant distortions in the protein-bound DNA datasets were observed in terms of unusual roll and twist values, which occur in DNA bound to a small group of protein families. Most of these protein-DNA complexes have been exhaustively studied because of their biological relevance, leading to the perception that protein-bound DNA structure very often differs significantly from free DNA structure. The protein-bound DNA structures that are observed to be distorted can be classified into three classes: the first where the DNA structure is distorted but the distortions do not lead to strand break or strand separation, the second where the distortion leads to a nick in the DNA backbone, and third where the distortion leads to strand separation. The first class consists of DNA bound by proteins belonging to the hyperthermophile and integration host factor families in the complex dataset, and to the CAP and lac repressor families in the HTH dataset. The second class consists of DNA bound to proteins belonging to the endonuclease family in the complex dataset, and to the transposase and recombinase families in the HTH dataset. The third class consists of DNA bound to polymerases and TBPs, and occur in both the bound DNA datasets. In the following two paragraphs, the structural features of these distorted DNA are described briefly.

We have classified a step as distorted if its roll or twist value deviates by more than 3*σ *from the mean roll and twist values of the free B-like DNA oligomer dataset. Additional file 3 gives the base-step parameters and Z_*p *_values for the distorted steps in the DNA structures bound to different protein families. It is clear that there are a wide variety of distortions in DNA structure, depending on specific family, to which the bound protein belongs. Most of the kinks lead to significant bending of the overall structure. In the CAP-DNA complexes (additional file 4, figure 1a) and the integration host factor-DNA complex (additional file 4, figure 1b), there is a nick in the DNA backbone that seems essential for the crystallization to succeed. It is quite likely that the presence of the nick facilitates the curvature of the DNA duplex, especially since other complexes (an ARAC family transcriptional activator-DNA complex (1BL0) and a CENP-B protein-DNA complex (1HLV)) with similar modes of protein-binding as that of CAP-bound DNA, display lesser degree of bending, as estimated by end-to-end bending angle as well as d/l_*local *_values (table 7). It might also be speculated that the presence of the nick allows the protein to distort the DNA at the local level to a greater extent, thus causing a few steps to assume unusually distorted roll and twist values, though, there is no direct evidence that this occurs.

**Table 7:** Curvature data for bound DNA duplexes of length ≥ 18 basepairs, excluding terminal basepairs, for the HTH-bound DNA dataset.

PDB ID	LENGTH	Succ. Bend. Ang. (°)				End-to-end (°) Bend. Ang.	d/l_*local*_	R.O.C.(Å)	RMSD (Å)			Geometry Assigned	Out-of-Plane (°) Bend. Ang.
		AVG.	S.D.	MAX.					Cfit	Lfit	Cfit/Lfit		
				Value	Position								
1KX5	30	15.1	10.5	51.9	A_10_A_11_A_12_	126.7	0.77	37.9	0.6	20.0	0.03	C	0.1

1J59	28	25.3	16.2	66.5	T_9_G_10_A_11_	98.5	0.85	47.2	0.7	21.4	0.03	C	-41.9

1RUN	28	30.4	17.1	71.1	T_9_G_10_A_11_	109.7	0.85	47.8	0.6	22.2	0.03	C	-50.6

1CGP	26	19.1	18.9	65.9	G_7_T_8_G_9_	72.4	0.86	48.2	0.8	17.9	0.04	C	-51.1

1BL0	20	10.5	6.1	25.0	A_6_G_7_C_8_	38.8	0.94	47.0	0.7	3.6	0.19	C	-68.9

1HLV	19	10.0	7.6	30.1	G_15_G_16_G_17_	39.3	0.95	54.6	0.6	3.3	0.18	C	-129.0

1APL	18	11.8	7.3	33.0	A_16_C_17_G_18_	3.7	0.99	NA	4.9	0.9	5.44	L	NA

1K78	23	9.1	3.2	15.6	T_19_G_20_G_21_	15.9	1.00	NA	2.4	1.0	2.40	L	NA

4CRX	32	11.8	12.2	56.5	A_17_T_18_G_19_	76.3	0.82	NA	3.4	9.1	0.37	U	NA

1GDT	32	15.4	13.0	60.3	T_14_T_15_A_16_	40.2	0.87	NA	1.6	14.5	0.11	U	NA

1MNM	23	11.8	7.8	26.8	G_13_A_14_A_15_	58.7	0.89	NA	1.7	5.4	0.31	U	NA

1JE8	18	23.0	13.0	49.6	T_2_A_3_C_4_	56.9	0.93	NA	1.1	5.0	0.22	U	NA

1DDN	23	8.8	4.4	19.1	T_13_T_14_A_15_	36.0	0.95	NA	7.7	2.5	3.08	U	NA

1L3L	18	9.0	5.9	19.7	C_14_A_15_C_16_	32.5	0.96	NA	1.5	1.6	0.94	U	NA

1U78	23	10.6	12.3	51.1	T_10_A_11_G_12_	41.3	0.97	NA	1.4	19.6	0.07	U	NA

1Z9C	24	10.0	5.9	23.9	T_11_A_12_T_13_	3.4	0.97	NA	6.1	2.0	3.05	U	NA

1H88	23	7.4	4.5	16.8	C_8_A_9_A_10_	12.3	0.98	NA	1.6	7.8	0.21	U	NA

1D5Y	18	8.9	5.7	21.6	C_15_A_16_A_17_	10.6	0.98	NA	2.6	2.0	1.30	U	NA

1K61	18	8.8	6.1	22.3	T_4_A_5_A_6_	6.6	0.99	NA	1.3	7.1	0.18	U	NA

1DU0	18	6.3	4.0	15.2	C_15_C_16_T_17_	10.1	0.99	NA	0.6	2.9	0.21	U	NA

1RIO	25	14.8	8.7	34.1	C_8_C_9_G_10_	16.1	0.99	NA	0.6	2.7	0.22	U	NA

6PAX	22	11.8	7.1	25.6	A_8_C_9_G_10_	20.4	0.99	NA	0.6	2.1	0.29	U	NA

2HDD	18	10.7	5.4	23.3	T_12_C_13_C_14_	22.9	0.99	NA	1.2	1.8	0.67	U	NA

1HDD	18	10.9	4.1	19.9	G_3_C_4_C_5_	1.9	0.99	NA	2.6	1.4	1.86	U	NA

3HDD	18	6.5	3.7	14.4	G_8_T_9_A_10_	8.3	0.99	NA	3.0	1.4	2.14	U	NA

1JT0	26	10.4	5.9	22.1	A_23_T_24_A_25_	29.8	0.99	NA	9.1	1.1	8.27	U	NA

1F4K	19	7.9	5.1	20.2	T_3_G_4_A_5_	24.8	1.00	NA	0.8	2.4	0.33	U	NA

1MDM	23	9.2	4.7	20.8	A_6_G_7_A_8_	15.6	1.00	NA	1.7	1.1	1.55	U	NA

1HF0	20	10.5	7.3	30.7	T_6_G_7_A_8_	28.6	1.01	NA	1.4	0.9	1.56	U	NA

The calculation of successive bending angles, end-to-end bending angle, d/l_*local*_, Radius of Curvature (ROC), RMSD for circle fit (Cfit) and line fit (Lfit) and torsion angle for out-of-plane component of bending have been described in the 'Methods' section. 'MAX.' denotes the position and value of the maximum successive bending angle within the particular structure. The criteria used to assign DNA molecule geometry as curved (C), linear (L) or unassigned (U) have also been described in the 'Methods' section. The radius of curvature (ROC) and out-of-plane component of bending are reported only when a DNA molecule's geometry is assigned as curved.

In most of these distorted DNA structures, the values of Z_*p *_and slide, as well as the backbone parameters retain classical B-DNA like values, indicating that B-DNA can accommodate considerable variations in roll and twist with little or no change in other parameters. Only a few steps in some of the endonuclease-bound DNA and the lac repressor-bound DNA have A-like Z_*p *_values. In these cases, one or two, and occasionally three of the four corresponding bases are observed to take up a C_3_'-endo sugar pucker. With the exception of a few endonuclease-bound DNA structures (1B94, 1B97, 1BGB, 1D02), the distorted steps themselves do not assume A-like Z_*p *_values, as evident from additional file 3. However, some of the distorted steps in the endonuclease-bound DNA have intermediate Z_*p *_values.

#### Gross structural level

Since the free DNA molecules are relatively short in length, it is difficult to ascertain whether the distortions observed at the local level add up to give a smooth global curvature. However both the protein-bound datasets contain several structures of greater length, hence we analysed the overall curvature of DNA structures from the complex and HTH datasets that consist of atleast 20 contiguous basepairs. We used the measures d/l_*local *_[63,65-68], the RMSD from circle fit and the ratio of the RMSD from circle fit to the RMSD from line fit, to characterise DNA curvature, as described in the 'Methods' section. Tables 7 and 8 give the values of the ROC for long duplexes (length ≥ 18 basepairs, excluding the terminal ones) in the protein-bound datasets. For the DNA duplex that is curved, we have also estimated whether the curved helix axis is planar or has an out-of-plane component. A segment of the nucleosome structure (1KX5 [86]) consisting of 30 basepairs gives a d/l_*local *_value of 0.77 and a ROC value of 37.9 Å, with circle fit standard deviation of 0.6 Å, indicating that the measures used are quite adequate to define curvature of DNA molecules of this length.

**Table 8:** Curvature data for bound DNA duplexes of length ≥ 18 basepairs, excluding terminal basepairs, for the protein (excluding HTH)-bound DNA dataset.

PDB ID	LENGTH	Succ. Bend. Ang. (°)				End-to-end (°) Bend. Ang.	d/l_*local*_	R.O.C.(Å)	RMSD (Å)			Geometry Assigned	Out-of-Plane (°) Bend. Ang.
		AVG.	S.D.	MAX.					Cfit	Lfit	Cfit/Lfit		
				Value	Position								
1KX5	30	15.1	10.5	51.9	A_10_A_11_A_12_	126.7	0.77	37.9	0.6	20.0	0.03	C	0.1

1KX3	30	19.3	9.9	37.4	G_3_C_4_A_5_	129.5	0.77	37.8	0.7	10.5	0.07	C	-4.7

1N3F	22	11.7	8.0	32.6	G_12_A_13_G_14_	23.4	0.97	65.1	0.5	3.2	0.16	C	-0.5

1T9J	22	13.3	9.3	32.0	C_6_G_7_T_8_	26.2	0.97	65.9	0.6	13.1	0.05	C	12.5

1G9Z	22	14.8	11.4	35.9	T_8_C_9_G_10_	22.9	0.97	68.3	0.6	13.0	0.05	C	-24.5

1T9I	22	12.1	9.3	28.6	C_6_G_7_T_8_	23.2	0.98	66.3	0.5	13.7	0.04	C	15.0

1OWF	20	16.9	15.5	56.7	T_11_T_12_G_13_	85.0	0.81	NA	1.2	13.2	0.09	U	NA

1CYQ	18	14.0	4.4	26.5	A_13_G_14_A_15_	55.0	0.90	NA	2.4	5.4	0.44	U	NA

1A73	18	14.1	4.5	23.0	A_13_G_14_A_15_	56.7	0.91	NA	2.3	5.3	0.43	U	NA

1ZS4	23	9.4	5.0	18.1	T_12_G_13_T_14_	8.1	0.99	NA	0.8	1.5	0.53	U	NA

1H6F	20	8.3	4.7	17.5	T_15_G_16_T_17_	26.2	0.99	NA	4.4	1.1	4.00	U	NA

The other specifications are as detailed in the caption to table 7.

Of the 28 HTH-bound DNA structures with length ≥ 20 basepairs (table 7), 7 meet the RMSD criteria that allows for reliable geometry assignment. Of these, 5 were found to have d/l_*local *_≤ 0.98. A further 21 duplexes that did not meet the RMSD criteria were classified as unassigned. Of the structures where the DNA is curved, the CAP-DNA complexes (1CGP, 1J59, 1RUN), an ARAC family transcriptional activator-DNA complex (1BL0) and a CENP-B protein-DNA complex (1HLV), all consist of a dimeric protein that binds to two successive major grooves of the DNA, approximately one helix turn apart and the DNA is essentially curved due to two major in-phase kinks (additional file 4, figure 1a). All these duplexes have a negative out-of-plane bending angle.

Among the 21 DNA duplexes that have been classified as unassigned, more complex types of protein-binding is observed, indicating that there are several different modes of curvature for a DNA bound to the HTH motif. In some of these structures (CRE recombinase protein (4CRX), *γδ *resolvase-DNA complex (1GDT)), bending appears to be a result of large kinks at one or two steps in the duplex, as is evident from the higher maximum bending angle values obtained for these structures.

Among the HTH-bound DNA duplexes that are found to be curved, only the CAP binding duplexes have large values for the average successive bending angle, while for all the other duplexes, it is < 12°, indicating that curvature of the duplex can arise due to the cumulative effect of small amount of bending along the entire helix. In contrast, some of the unassigned duplexes that have d/l_*local *_> 0.98, and so could be considered linear, were observed to have large average successive bending angle values (lambda CI-NTD – sigma-region4 – DNA complex (1RIO, shown in additional file 4, figure 1c), PAX5-DNA complex (6PAX)), indicating that large distortions at the local level can cancel each other out and so need not cause the entire duplex to bend significantly.

**Figure 6:**
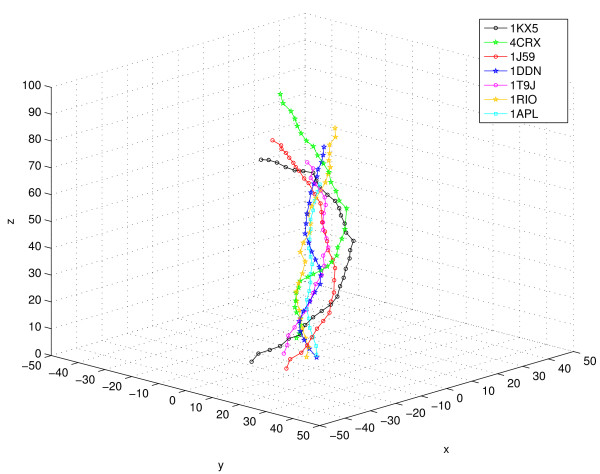
**The 3-dimensional path traced by basepair centers of the DNA helix, in some protein-DNA crystal structure complexes**. The basepair centres of the DNA molecules are indicated by hollow circles in case of 'curved' geometry, by hollow squares in the case of 'linear' geometry and by stars in case of 'unassigned' geometry. The criteria for assigning geometry has been described in the 'Methods' section. The PDB id's correspond to the following biological molecules: 1KX5 – Nucleosome core particle, 4CRX – CRE recombinase protein-bound DNA, 1J59 – Catabolic Activator Protein (CAP)-bound DNA, 1DDN -Diphtheria tox repressor-bound DNA, 1T9J – Endonuclease-bound DNA, 1RIO – lambda CI-NTD-sigma-region4-bound DNA, 1APL – MAT alpha2 homeodomain-bound DNA.

In the complex dataset, there are only 9 structures (excluding the reference nucleosome structure) with DNA length ≥ 20 basepairs (table 7). Of these, 4 were found to be curved by the criteria of d/l_*local *_and ROC. The curved duplexes all comprise of DNA bound by I-Cre I endonucleases that bind to and have interactions along the entire length of the duplex. Among the unassigned duplexes, the DNA assumes a U-shaped structure in the integration host factor-DNA complex 1OWF (additional file 4, figure 1b), wherein a nick has been introduced into the DNA backbone to facilitate crystallisation. The duplex upto the nick has a d/l_*local *_value of 0.81 (given in table 8), but the entire 34 basepair duplex has a d/l_*local *_value of 0.33. In two endonuclease-bound complexes 1A73 and 1CYQ, the presence of junctions between two A-like regions separated by a B-like region leads to a non-linear geometry, as indicated by the d/l_*local *_values (~0.90), but the duplexes are not smoothly curved. A lambda-CII-DNA complex (1ZS4), and a human TBOX-protein 3-DNA complex (1H6F), are linear as per d/l_*local *_values. Some representative examples, showing the 3-dimensional path of the basepair centres in the DNA duplex, with different amounts of curvature are shown in figure 6, while the cartoon diagrams of few DNA-protein crystal structure complexes are shown in additional file 4, figure 1. They clearly illustrate the different extent of curvature (or lack of it), adopted by protein-bound DNA molecules.

No correlation was observed between the curvature and occurrence of the various backbone geometries (even the energetically unfavourable state 6 conformation) in these structures.

## Discussion

From our analysis, it is clear that for the individual dinucleotide steps in the free oligomer dataset, the dinucleotide step parameters Z_*p *_and to a lesser extent, slide, as well as the pseudorotation phase angle P for the sugar ring, the backbone torsion angle *χ *and the glycosidic torsion angle *δ *for the individual bases in a step are better indicators of A-like or B-like conformation than the traditionally used parameters of roll and twist, confirming the findings by earlier studies [14,27]. A few of the dinucleotide steps seem to have a distinct preference for a particular conformation-AA and GA steps are strongly B-philic, while only the GG step is strongly A-philic.

In an earlier study to characterize how the DNA sequence defines conformation, Hays et al [16] have reported crystal structures of all the permutations of the inverted repeat d(CCnnnN_6_N_78_GG) under well-defined crystallographic conditions, which take up A-form, B-form and Holliday junction structures. Several of the structures reported in that work fit our selection criteria and are also part of the free dataset in the present study. The authors observed that the set of A-DNA crystal structures reported in their study are conformationally more uniform than the B-DNA structures. This also seems to be the case for the larger and sequentially more heterogeneous dataset analysed in this study vis-à-vis Z_*p*_, slide, roll and twist, not only for the entire free dataset, but also for the individual basepair dinucleotide steps. With the exception of the slide parameter for AT step, and the roll parameter for GG step, the standard deviation is always higher for the B-like free DNA steps as against the A-like steps, for all the four parameters. However, for a step like CA, one must consider the fact that it is in fact trimodal, with the B-like steps further subdivided into BI and BII conformations, making it difficult to compare the variation between A-like and B-like conformations. It must also be noted that with the exception of slide in case of A-like steps, the standard deviations obtained in our study are lower compared to those observed by Hays et al [16] for the slide, roll and twist parameters examined in both the studies. Hays et al [16] observed that the trinucleotide motifs GGN, NGG and CC(C/G) favour a transition to A-DNA conformation. Our analysis supports this conclusion, with most of the GC rich structures taking up an A-DNA conformation. The only exception to this rule was observed to be 1ZFB, for the sequence d(CCGCCGGCGG). However, an earlier structure (382D [88]) of the same sequence (not included in this study owing to resolution cutoff criteria), was observed to have an A-like conformation. Hence A-DNA definitely seems to be favoured by GC rich DNA, especially those with oligo-G tracts. Since the GG step is observed to be the most A-philic, this is to be expected. However a GC rich sequence which does not have an oligo-G tract does not necessarily favour an A-DNA conformation, since GC and CG steps seem to assume A-like and B-like conformation with nearly equal ease. In addition, with the exception of AC, which seems to equally favour the A and B-form, and CA, which is trimodal, all other steps where one or both basepairs are A:T seem B-philic in the free dataset.

For B-DNA structures, Gorin et al [11] have correlated the extent of B-DNA twisting with the basepair morphology and clash between the exo-cyclic groups in the four bases. The average values for slide, roll and twist, obtained by Gorin et al [11] for the dinucleotide steps in their dataset, comprising of B-DNA structures with a resolution cutoff of 3.0Å, are quite similar to the 'B-like' average values for different dinucleotide steps in the present study. (tables 3, 4, 5). The overall average values in the two studies are also observed to be similar. However, interestingly the low twist CA (BI) and high twist TA steps in Gorin et al dataset converge to nearly similar values in our high resolution dataset (36.5° and 34.7° respectively). This positions the CA step in a favoured conformation with minimal clash as predicted by the clash strength function designed by these authors, while the TA step is positioned in a less favourable conformation.

### How different is bound DNA from free B-DNA?

In case of bound DNA, the DNA duplexes are almost entirely B-like in conformation in terms of Z_*p *_and slide, while roll and twist predominantly show variation that is similar to that of free DNA. The average values for slide, roll and twist, obtained in an earlier study by Olson et al [25] for different dinucleotide steps in a dataset of protein-DNA crystal structure complexes, are comparable to those obtained in this study for the complex dataset (excl. TEH, tables 3, 4, 5) and the HTH dataset (excl. TC, tables 3, 4, 5). This is expected, since these authors also considered only the step parameter values within 3*σ *deviation of the mean, for their 'B-like' protein-DNA dataset, essentially excluding the distorted steps. The overall average values for slide, roll and twist reported for the 'B-like' protein-DNA dataset are also observed to be similar to those obtained for the complex (excl. TEH) dataset and the HTH (excl. TC) dataset in this study.

Only a few DNA structures, bound to proteins belonging to a small group of families have highly unusual structural parameters, principally roll and twist. Apart from those structures, other structures in both the protein-bound datasets principally take up free B-DNA like values for all the dinucleotide step parameters. The nucleosome structure [86], considered a classic case of a highly curved structure, does not have highly unusual Z_*p*_, slide, roll or twist values, with only 5 out of 146 roll values lying just outside the 3*σ *deviation range of the B-DNA like oligomer dataset. The lack of sharp kinks gives the nucleosome structure a smooth curvature, with very small out-of-plane component for ~30 basepair fragments. For any randomly selected 30 basepair fragment of this structure, the RMSD from a plane fit was always < 0.15 Å, as against a value of 0.65 Å for the highly distorted CAP-bound DNA structure 1J59. Even for a randomly selected fragment of 76 basepairs (which nearly completes a full circle), the RMSD from a plane fit was observed to be only 0.26 Å. Similarly, the out-of-plane torsion angle values, for several random fragments of ~30 basepairs, were always observed to be < 10°, another indicator of the smooth curvature and gentle, regular pitch of the superhelix. It is also interesting to note that the ROC calculated for the 76 basepair fragments in 1KX3 and 1KX5 are 39.8 Å and 39.4 Å respectively, while that for a 2.8 Å resolution structure (1AOI [89]) is calculated as 41.5 Å, indicating that the DNA in different nucleosome structures has small variations in curvature.

#### A-like steps are limited to DNA bound to proteins from a few specific families

Protein-bound DNA structures, apart from being perceived as distorted, have also been characterised as being predominantly A-like [90]. Our analysis clearly refutes this characterisation. The only protein-bound DNA structures having few steps with A-like values of Z_*p *_are those bound to some of the endonucleases, DNA polymerases, transposases and the homeodomains. Of the endonuclease bound-DNA structures, the IPpo I endonuclease-bound DNA structures 1A73 and 1CYQ have two separate A-like half turns [14], leading to a non-linear geometry, as explained above. The PvuII endonuclease-bound DNA structure 3PVI has an entire A-like stretch with only few bases at one end having a B-like geometry in terms of Z_*p*_.

Polymerase-bound DNA (1L3S, 1L3U, 1L3V, 1L5U, 1L3T, 1LV5, 1NJY, 1NJZ, 1NK5, 1NK6, 1NK7, 1NKC, 1U45, 1UA1, 2BDP, 4BDP) undergo gradual transition in Z_*p *_and slide, with B-like values at one end to intermediate to A-like values towards the nucleotide incorporation end, but do not show large variation in their B-like roll and twist values. At least two, and in several instances three, of the values for P, *δ *and *χ *are A-like. The DNA duplexes in these complexes have a variety of sequences indicating that the A-like nature of these duplexes is not sequence dependent, but is a result of protein-binding and the polymerisation process itself.

Transposase-bound DNA (1TC3, 1U78) have a 4–7 basepair long G:C rich region at one terminal of the duplex that assumes A-DNA like conformation in terms of all the parameters. The other end of the DNA duplex is A:T rich, with a narrow minor groove [91] and curvature characteristic of DNA containing oligo A-tracts [58,59]. The transposase protein binds to these two regions-the G:C rich A-like region and the A:T rich region, via two HTH motifs that are connected by a long linker [91]. However, the features observed for the G:C rich region and the oligo A-tract in the transposase-bound DNA are similar to those observed for free G:C rich oligomers and free oligo A-tracts, hence these features can be said to be intrinsic to the DNA sequence.

There are 18 homeodomain-bound DNA structures in the protein-bound datasets. Of these, only five steps (3 GG and 2 CG steps), occurring in four different structures, are observed to take up A-like values of Z_*p*_.

Another class of DNA, often cited as an example of protein-induced B ↔ A transition, are the Zn-finger-bound DNA structures. Nekludova et al [92] have shown that for a variety of protein-bound DNA molecules, including Zn-finger-bound DNA, a distinctive conformation with an enlarged major groove when compared to B-DNA, was observed. In our study, all the Zn-finger-bound DNA structures (1A1G, 1A1H, 1A1I, 1A1J, 1A1K, 1A1L, 1AAY, 1G2F, 1JK1, 1JK2, 1LLM) (see additional file 1 for the references for all these structures) assume B-like values of Z_*p *_for all the steps. Similarly the backbone sugar pucker and the torsion angle *χ *are observed to be close to B-DNA fibre model values. Several other parameters, indicating the A-like or B-like nature of a DNA structure, also assume B-like or intermediate values. The average global helical rise (3.3 ± 0.1Å) is entirely B-like. The slide values are B-like to intermediate, whereas the average values of global x-displacement (-1.4 ± 0.5 Å), inclination (7.1 ± 4.7°) and helical twist (33.5 ± 4.9°) are intermediate but closer to B-DNA fibre model values. The average interstrand P-P distance across the major groove is 18.0 ± 1.6 Å, very close to the B-DNA fibre model value while the average interstrand P-P distance across the minor groove is 12.6 ± 1.3 Å, again only marginally larger than the B-DNA fibre model value.

It is also interesting that while the 7 nucleotide long runt-domain binding site DNA sequences in the free form are reported to assume A-DNA like conformation (1XJX, 1XJY) [34] as well as near B-DNA like conformation [34,93], depending on the flanking bases, the same sequences bound to the runt-domain protein are found to assume B-like values of Z_*p*_, slide, the backbone torsion angles and the groove widths. X-displacement, inclination and helical twist take up intermediate, but closer to B-DNA values – a behaviour similar to that observed for the bound DNA datasets in this study. Thus, in this case, while the free DNA sequences assume both the A and B-forms, the protein-bound DNA take up the B-form.

It has been suggested that the TBP-bound DNA conformation is closer to an A-DNA and the inherent A-philicity of the TATA sequence might facilitate the transition to the near A-like bound-conformation [39]. Our analysis indicates that these assertions are not always valid. For example, the oligomer structure 1VJ4 [50] for the sequence d(GGTATACC), takes up an A-DNA conformation, but the free DNA structures 1D56 and 1D57 [81], for the decamer d(CGATATATCG), both take up a B-DNA conformation, despite encompassing the TATA stretch. The TATA stretch in the TBP-bound DNA structures also take up entirely B-like Z_*p *_values. Though some of the other parameters such as roll, twist and rise do not have classical B-DNA values, this is more indicative of a distortion from the B-form, but not necessarily to an A-like conformation. The B-like nature of the TBP-bound DNA in terms of Z_*p *_and slide is also observed for a couple of hexamer sequences, which occur in both the free DNA dataset and some of the TBP-bound DNA structures. While the sequence TTTAAA takes up B-like Z_*p *_and slide values in the free (1IKK [94], 1SK5) as well as the TBP-bound DNA (1D3U [95], 1QNA [96]), the hexamer stretch GGCGCC takes up an A-DNA conformation in the free DNA structure 414D [97] as expected, but is observed to take up B-like Z_*p *_and slide values in the TBP-TFIIB-DNA complex 1C9B [98]. It is also noteworthy that unlike the TBP-bound DNA from the complex dataset, the backbone parameters P and *δ *as well as *χ *take up entirely B-like values for the TBP-TFIIB-bound DNA from the HTH dataset, consistent with our observation throughout this study that HTH-bound DNA tends to be more B-like than other protein-bound DNA molecules.

There have been studies of protein-DNA complexes, using backbone conformational parameters such as sugar pucker [99] or the *χ *and *δ *torsion angles [90] to classify the DNA nucleotides as A-like or B-like. The Z_*p *_versus sugar pucker (figure 3) plot as well as the Z_*p *_versus *δ *(additional file 2, figure 2) and Z_*p *_versus *χ *(additional file 2, figure 3) plots clearly indicate that a C_3_'-endo conformation or A-like values of *χ *or *δ *do not necessarily imply an A-like conformation in the protein-bound datasets. Significantly, Tolstorukov et al [99] find only 12% of the protein-interacting nucleotides with a C_3_'-endo sugar pucker conformation. On the other hand, Lejeune et al [90] conclude that "A-DNA is more frequently implicated in protein-DNA interactions than the classical B-DNA conformation". We do not find this claim to be valid, using any of the backbone parameters for 'A versus B' discrimination.

#### HTH-bound DNA, while remaining B-like, occassionally takes up an unfavourable backbone conformation

The only effect that can be unambiguously ascribed to protein binding in the predominantly B-DNA like protein-bound duplexes occurs in the DNA backbone. The DNA backbone in the free dataset is quite uniform, with the angles *α *and *γ *almost completely in the canonical *g*^-^, *g*^+ ^conformation in B-DNA, and (*α*, *γ*, *ε*-*ζ*) ranging from (*g*^-^, *g*^+^, BI) to (*t, t*, BI) conformation in A-DNA. On the other hand, backbone torsion angles in protein-bound DNA are observed to be considerably distorted. Steps that are B-like in terms of Z_*p *_and slide are observed to assume a wide variety of backbone conformations that are highly unusual, and in some cases, energetically unfavourable. In particular, HTH binding causes *α *and *γ *angles in DNA to assume the energetically unfavourable *g*^+^, *g*^- ^conformation in much higher proportion (11.7%) than in unbound DNA. As described in the 'Results' section, the steps taking up this energetically unfavourable conformation occur with equal frequency in the bound as well as the unbound regions of the DNA, and are not observed to assume unusual values for any other structural parameter. Overall, 57 out of the 97 HTH-motif bound DNA structures are observed to adopt this unfavourable backbone conformation for some of the steps. Of these, 24 structures have 5 or more occurrences of the unfavourable backbone conformation. Thus it is seen that there are a large number of structures with atleast a few steps in this conformation. These structures have been solved in a variety of space groups. The proteins binding to the 24 HTH-bound DNA structures with 5 or more occurrences of state 6 get classified into 15 different SCOP classes. Thus it appears that binding by the HTH motif allows the DNA backbone to assume this energetically unfavourable conformation, even when there is no direct contact between them.

At the tri-nucleotide level, bound DNA, and especially HTH-bound DNA appears to have less distortion than free DNA. At the gross structural level, nearly half of the DNA structures of length ≥ 20 basepairs and bound to the HTH motif were observed to have moderate curvature. It was observed that in several of these cases, the DNA was bound by a dimer of 2 HTH motifs, with the two monomers binding to DNA at regions one helix turn apart and bending it in the same direction so that there was a net overall curvature. However there are other modes by which the DNA bound to the HTH motif was observed to be curved, such as in the case of the MAT alpha2-MCM1-DNA ternary complex 1MNM and the CRE-recombinase-DNA complex (4CRX). Yet other modes of curvature of protein-bound DNA are revealed in the complex dataset. Thus it is not possible to determine a uniform mode and mechanism for the DNA curvature observed in the bound datasets. With the exception of a few structures where it was difficult to determine three uniformly undistorted regions separated by a large kink, all the curved DNA structures have a negative out-of-plane component. With no long free DNA oligomers in the dataset, it is difficult to conclude whether free DNA by itself can attain such conformations and the protein merely 'locks' it in that conformation or the protein actually bends it to that state. Most of these curved structures, however, do not have highly unusual step parameter values and hence it is possible that longer free DNA oligomers with similar sequences might be able to achieve such curved conformations without the aid of proteins. Even a few steps with unusual parameters might occur in long free DNA oligomers, as indicated by the spontaneous development of one or two sharp kinks in the molecular dynamics simulations of 94 basepair free DNA minicircles [100]. This has interesting implications especially for the HTH-binding DNA, since a majority of the proteins in this dataset are transcription activators or repressors, whose function on binding to the DNA is to cause structural changes in the DNA that allow or prevent other proteins of the transcription machinery to bind to the DNA and carry out transcription. It is tempting to speculate that these proteins merely increase the 'lifetime' of those conformations, as against inducing unfavourable conformations, which involves a much higher energetic cost. However, this needs to be verified using experimental and theoretical methods that trace the dynamic evolution of DNA structures under different conditions.

## Conclusion

The free DNA oligomers, even in the crystalline state, sample a large conformational space, but each molecule is found to be entirely in the A or B form, depending primarily on its sequence. In case of protein-bound DNA, the claim that protein-binding generally favours the A-form of DNA [90], as well as the perception that it induces an energetically unfavourable conformation, are invalid. We find that the role of A-form is limited to the DNA structures bound to a few specific protein families such as transposases and DNA polymerases. Protein-induced distortion in DNA can occur via one of several different modes, such as a few steps taking up high positive roll and a smaller twist, a BII like transition of the backbone, leading to a negative roll and large twist, or in some cases, the two strands in the helix being pulled apart. However, these large, induced deviations from the free B-form are observed only in the DNA structures bound to the proteins such as CAP, TBP, integration host factor and Cre recombinase. It is to be noted that, even in these structures, the distortions are limited to a few steps and the remainder of the duplex shows B-DNA like features. In a large number of cases of the HTH motif-bound DNA, protein-binding does not induce any distortion in the dinucleotide step geometry, but the duplex takes up an energetically unfavourable backbone conformation, even when there are no contacts between the protein and the DNA backbone. Barring these exceptions, the average parameters at the level of dinucleotide step, trinucleotide and the backbone of protein-bound DNA structures, across a large and diverse set of protein families, are quite close to the free B-DNA oligomer values. Interestingly, this is observed even though very few hexamer or longer sequence motifs are common to the free and bound datasets, and the free DNA dataset is significantly smaller than the bound DNA datasets in terms of size. It is also striking to note that even a duplex structure as far away from a 'straight' DNA as seen in the 147 basepair long nucleosome, has very few (≤ 5) steps with highly distorted local parameters, indicating that 'normal' B-like parameters at the local level can cumulatively give rise to double helical structures with a wide range of geometries. These observations highlight the amazing adaptability of this structural form, and may explain why it has evolved to be biologically the most relevant design for double-helical DNA.

## Methods

### Crystallographic dataset generation

The four X-ray crystallographic datasets used in the analysis are (i) RNA oligomers dataset (hereafter referred to as the RNA dataset), (ii) DNA oligomer dataset (hereafter referred to as the free dataset), (iii) DNA-protein complexes dataset (excluding DNA bound by the HTH protein) (hereafter referred to as the complex dataset), and (iii) DNA-HTH protein complexes dataset (hereafter referred to as the HTH dataset). The RNA, free and complex datasets were extracted from the Protein Data Bank (PDB) [101]. All three datasets contain structures with a resolution of 2.0 Å or better. Structures in the PDB that have the DNA-binding HTH motif were identified using the tool PredictDNAHTH, developed by McLaughlin et al [102]. Since only 33 DNA-HTH protein complexes with a resolution of 2.0 Å or better were identified, the resolution cut-off for the HTH dataset was increased to 3.0 Å. There was no significant difference between the results obtained for the dataset with a cutoff of 2.0 Å and the dataset with a cutoff of 3.0 Å. Therefore the larger dataset, with a cutoff of 3.0 Å was used. In the three DNA datasets, only fragments of the DNA consisting of atleast 8 contiguous Watson-Crick basepairs were considered. The RNA dataset had much shorter structures, hence the length cutoff was reduced to five contiguous basepairs. Also steps with non-Watson-Crick basepairs, present in significant numbers in the RNA dataset, were not included in this analysis. In the free dataset, structures with any ligands other than ions or water were excluded. Identical basepairs from structures with a two-fold symmetry were considered only once. The RNA dataset consists of 52 structures (additional file 1) (75 individual duplexes which contain 276 dinucleotide steps comprising of Watson-Crick base pairs). The free dataset consists of 76 structures (additional file 1) (77 individual duplexes which contain 406 basepaired dinucleotide steps comprising of Watson-Crick basepairs).

The complex dataset consists of 85 structures (additional file 1) (112 duplexes which contain 1227 dinucleotide steps comprising of Watson-Crick basepairs). The HTH dataset (at ≤ 3.0 Å) consists of 97 structures (additional file 1) (126 duplexes which contain 1559 dinucleotide steps comprising of Watson-Crick base pairs).

### Evaluation of dinucleotide step parameters and global helical parameters

The structural parameters of the duplexes i.e. the basepair parameters propeller twist, buckle, opening angle, shear, stretch and stagger as well as the dinucleotide step parameters tilt, roll, twist, shift, slide, and rise were determined by the NUPARM program [103-105], for all the four datasets. The parameter Z_*p *_[14], defined as the mean z-coordinate of the backbone phosphate atoms of the basepair with respect to the basepair dimer reference frame, was also calculated using the revised NUPARM program [105].

The dinucleotide step parameters tilt, roll and twist measure the relative rotational motion between adjacent basepairs about the x, y and z-axis respectively of a local basepair doublet coordinate system, whereas the dinucleotide step parameters shift, slide and rise measure relative translational motion between adjacent basepairs along the local doublet x, y and z-directions respectively.

The global helical parameters viz. the rotational parameters inclination, tip and the helical twist and the translational parameters x-displacement, y-displacement and z-displacement were also calculated using the NUPARM program. Inclination denotes the rotation of the basepair about the x-axis, tip denotes rotation about the y-axis and helical twist denotes rotation about the helical axis. Similarly the translational parameters denote displacement along the three axes. The mean of the global x-displacement, helical rise, inclination and helical twist for all the non-terminal basepairs within all the structures in a dataset were classified as the average values for the respective dataset. The protein-bound DNA sructures in which the roll or twist value for atleast one step deviated by more than 3*σ *from the mean roll and twist values of the free B-like DNA oligomer dataset, and also those structures which were curved or whose geometry of curvature could not be assigned (as given in tables 7, 8), were excluded from the calculation of mean values of global helical parameters, since fitting a single linear helical axis would be untenable in these cases. Overall, 49 structures from the complex dataset and 62 structures from the HTH dataset were included for these calculations.

### Evaluation of groove widths

The minor groove width and the major groove width were calculated as the smallest interstrand phosphate separations along the two grooves, using the NUPARM program. Please note that the groove widths as defined here also include the phosphate diameter value.

### Calculation and classification of backbone torsion angles

Backbone torsion angles *α*, *β*, *γ*, *δ*, *ε*, *ζ*; the glycosidic torsion angle *χ *and the pseudo rotation angle P [1] were calculated using the NUPARM program. Backbone torsion angles for a basepaired dinucleotide step, i.e., across the phosphodiester bond, were clustered and analysed. *ε*: C4'_*n*_-C3'_*n*_-O3'_*n*_-P_*n*_, *ζ*: C3'_*n*_-O3'_*n*_-P_*n*+1_-O5'_*n*+1_, *α*: O3'_*n*_-P_*n*+1_-O5'_*n*+1_-C5'_*n*+1 _and *γ*: O5'_*n*+1_-C5'_*n*+1_-C4'_*n*+1_-C3'_*n*+1 _are classified into seven states as per the algorithm proposed by Dixit et al [53]. Since *ε *and *ζ *assume two related conformations, *ε*, *ζ *= t, g^- ^being the canonical conformation, known as BI, and *ε*, *ζ *= g^-^, t being the non-canonical conformation, known as BII, a value of *ε *- *ζ *≤ 0 has been classified as the BI conformation, and a value of *ε *- *ζ *> 0 has been classified as the BII conformation.

### Calculation of bending/curvature

The calculation of the radius of curvature using a least square circle fit method and the ratio of end-to-end distance to the contour length (d/l_*local *_or d/l_*max*_) were done as described previously in [69]. The measure d/l_*max *_is reasonably independent of the length of the DNA sequence (data not shown), except for highly curved long DNA molecules, as in nucleosomal DNA, but does not distinguish between different types of bending for sequences with fewer than 30 basepairs (data not shown). The radius of curvature (ROC) is calculated by fitting a circle to the basepair centres of the DNA molecules. Smaller the radius of this circle, the more curved the DNA is. However, the quality of the fit to a circle is affected to a large extent by distortions at the local level in the duplex i. e. the successive bending angles. Thus the presence of several triplets that are distorted, even to a small degree, would lead to a poor circle fit and consequently an inaccurate value of radius of curvature (ROC). Thus ROC is only reported when the RMSD for a circle fit is ≤ 1.0 Å, and the ratio of RMSD for a circle fit to that for a line fit is ≤ 0.6.

When the d/l_*local *_value is ≤ 0.98, the RMSD for a circle fit is ≤ 1.0 Å, and the ratio of RMSD for a circle fit to that for a line fit is ≤ 0.6, we have assigned the DNA molecule geometry to be curved. When the d/l_*local *_value is > 0.98, the RMSD for a line fit is ≤ 1.0 Å, and the ratio of RMSD for a circle fit to that for a line fit is > 1.6, we have assigned the DNA molecule geometry to be linear. When neither the 'curved' nor 'linear' criteria are satisfied, the geometry of the DNA duplex is considered as 'unassigned'. For the DNA duplex that is curved, the out-of-plane component of DNA curvature was calculated as the torsion angle between the global helix axes vectors fitted to three relatively straight sections of the DNA molecule, separated by large kinks.

A local helix axis vector corresponding to each dinucleotide step is defined as the vector pointing in the direction of the cross-product of the differences of the x and y-vectors of the constituent basepair planes. The angle between two local helix axes vectors corresponding to overlapping dinucleotide steps, described as the successive bending angle, as well as the angle between the vectors corresponding to the dinucleotide steps at the two ends of the molecule, and described as the end-to-end bending angle, were also calculated using NUPARM and used as measures of curvature.

The entire analysis of the dinucleotide step parameters, backbone torsion angle parameters, the successive bending angles, the radius of curvature, d/l_*local *_and out-of-plane components of DNA curvature has been carried out excluding the terminal basepairs to eliminate end effects. The end-to-end bending angle has also been measured as the angle between the local helix axes vectors corresponding to the penultimate dinucleotide steps.

All the plots were generated using the MATLAB-7.4 package.

The values of the basepair parameters, base-step parameters as well as the backbone torsion angles obtained using the NUPARM package were compared to those obtained by the X3DNA package [27]. The general trend of the parameters was observed to be similar. The parameters calculated by the two programs were different for the distorted regions of a few protein-bound DNA structures.

## Authors' contributions

AM and MB conceived the study. AM developed the various tools for analysis and carried out the analysis of the free and complex datasets. DK carried out the analysis of the HTH-bound dataset. AM, MB and DK drafted the manuscript.

## Supplementary Material

Additional file 1 **PDB id's and corresponding references for the X-ray crystal structures used in this study**. This file contains the PDB id's of the crystal structures and the citation for each of these structure. Click here for file

Additional file 2 **Variation of different local and global parameters for the four datasets**. This file contains three figures and a table. The figures show the correlated variation of different parameters, namely; twist versus roll, Z_*p *_versus backbone torsion angle *χ *and Z_*p *_versus backbone torsion angle *δ*, for the four datasets used in this study. The table shows the values of the global helical basepair orientation parameters and the minor and major groove widths for the four datasets. Click here for file

Additional file 3 **Parameters for the distorted dinucleotide steps**. This file gives the dinucleotide step parameters and Z_*p *_values for the distorted steps in the DNA structures bound to different protein families. Click here for file

Additional file 4 **Cartoon diagrams of few DNA-protein crystal structure complexes**. This file contains the cartoon diagrams of four DNA-protein X-ray crystal structure complexes with different amounts of curvature. Click here for file
