# Supplementary material for: Small local variations in B-form DNA lead to a large variety of global geometries which can accommodate most DNA-binding protein motifs
Source: BMC Struct Biol. 2009 Apr 24;9:24. doi: 10.1186/1472-6807-9-24 (PMC2687451; doi:10.1186/1472-6807-9-24)
Supplement: Additional file 2 — Variation of different local and global parameters for the four datasets. This file contains three figures and a table. The figures show the correlated variation of different parameters, namely; twist versus roll, Zp versus backbone torsion angle χ and Zp versus backbone torsion angle δ, for the four datasets used in this study. The table shows the values of the global helical basepair orientation parameters and the minor and major groove widths for the four datasets. [file 1472-6807-9-24-S2.pdf]

Variation of different local and global parameters

for the four datasets

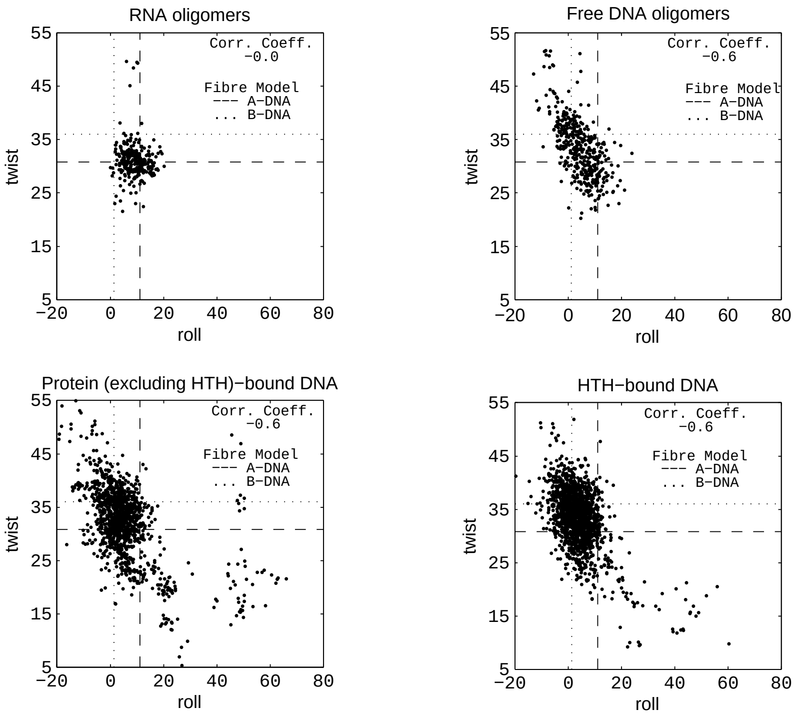

Figure 1: Twist versus Roll for the RNA, free DNA oligomers, protein (excluding HTH)-bound DNA and HTH-bound DNA datasets. Dashed and dotted lines have been drawn to highlight the values for the A and B-DNA fibre models.

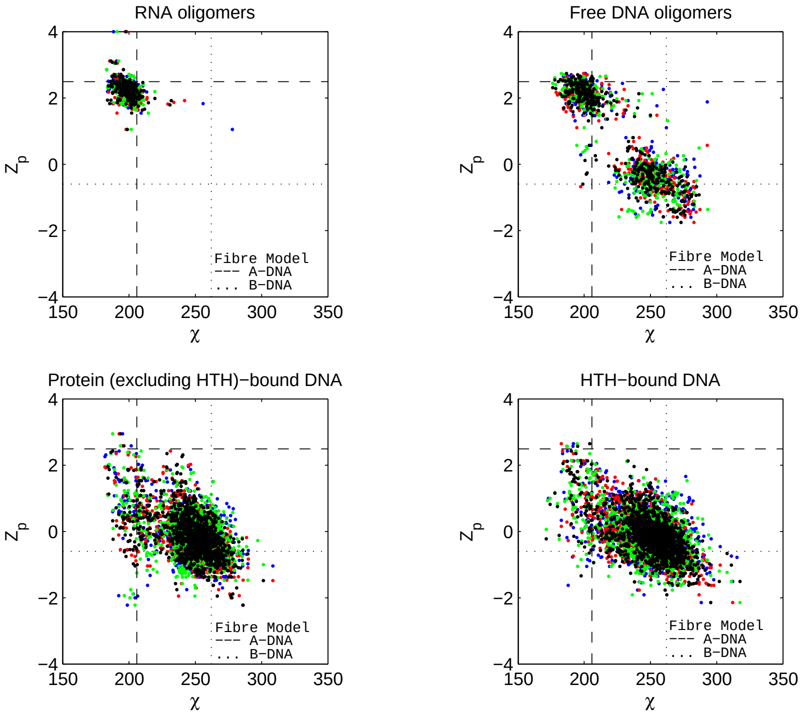

Figure 2: Z_p versus backbone torsion angle χ for the four datasets. The four nucleotides constituting a basepaired dinucleotide step corresponding to a particular Z_p value have been colour coded as follows: *5'-end Strand 1 - blue; 3'-end Strand 1 - Red; 5'-end Strand 2 - Green; 3'-end Strand 2 - Black.*

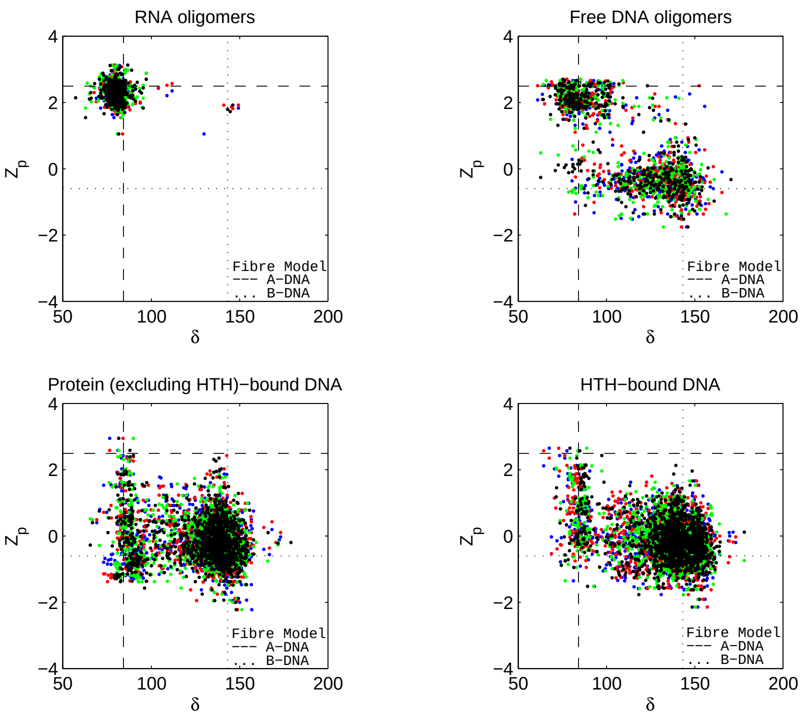

Figure 3: Z_p versus backbone torsion angle δ for the four datasets. The four nucleotides constituting a basepaired dinucleotide step corresponding to a particular Z_p value have been colour coded as follows: *5'-end Strand 1 - blue; 3'-end Strand 1 - Red; 5'-end Strand 2 - Green; 3'-end Strand 2 - Black.*

Table 1: Mean and standard deviation values (in parentheses) for global helical basepair orientation parameters and groove widths. The linear global helical axis has been fitted to the backbone C_1' atoms. For the complex and HTH datasets, structures which are curved or whose geometry of curvature could not be assigned are excluded, since a linear global helical axis cannot be fitted to such structures.

	Fibre-model		RNA	Free		Complex	HTH
	A-DNA	B-DNA		A-like ($Z_p > 1.3$ Å)	B-like ($Z_p \leq 0.8$ Å)	Excl. TEH†	Excl. TC ‡
Inclination (°)	19.3	2.0	15.4 (4.8)	16.5 (5.1)	2.0 (3.5)	6.4 (6.2)	5.9 (6.5)
Helical twist (°)	32.7	36.0	32.7 (6.1)	32.1 (3.7)	35.9 (6.0)	34.1 (4.6)	34.0 (4.8)
X-displacement (Å)	-4.5	0.6	-4.3 (1.2)	-4.3 (0.9)	0.1 (0.8)	-1.1 (1.1)	-0.9 (1.3)
Helical rise (Å)	2.6	3.4	2.7 (0.3)	2.7 (0.3)	3.3 (0.1)	3.2 (0.1)	3.2 (0.2)
Smallest P-P distance (Å) (across minor groove)	17.0	11.7	15.7 (0.6)	15.8 (0.6)	11.1 (1.5)	12.4 (1.7)	12.5 (1.8)
Smallest P-P distance (Å) (across major groove)	8.2	17.2	11.1 (2.0)	11.6 (2.7)	17.1 (0.9)	17.3 (1.7)	17.1 (1.3)

†TEH - TBP, Endonuclease and Hyperthermophile chromosomal protein SAC7D containing structures (excluded)

‡TC - TBP and CAP containing structures (excluded)
