# Supplementary material for: Small local variations in B-form DNA lead to a large variety of global geometries which can accommodate most DNA-binding protein motifs
Source: BMC Struct Biol. 2009 Apr 24;9:24. doi: 10.1186/1472-6807-9-24 (PMC2687451; doi:10.1186/1472-6807-9-24)
Supplement: Additional file 4 — Cartoon diagrams of few DNA-protein crystal structure complexes. This file contains the cartoon diagrams of four DNA-protein X-ray crystal structure complexes with different amounts of curvature. [file 1472-6807-9-24-S4.pdf]

Cartoon diagrams of few DNA-protein crystal structure complexes

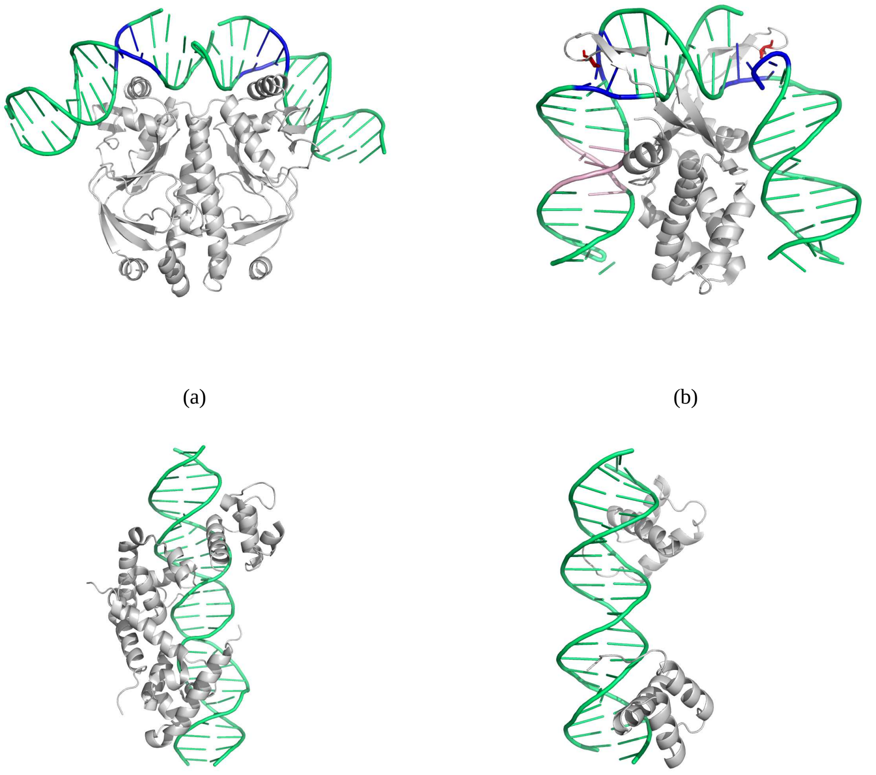

(a) (b)

(c) (d)

Figure 1: DNA-protein complexes showing different modes of protein-induced distortions and curvature (or lack of it) (a) 1J59 - CAP-DNA complex, where the DNA is curved via two kinks that occur about 10 basepairs apart; (b) 1OWF - integration host factor-DNA complex, where the intercalation of two proline residues at steps about 10 basepairs apart kinks the DNA and causes it to assume a U-like structure. Intercalating proline residues in 1OWF are shown in red. The CAA triplet in the DNA in 1OWF, which is the specific protein binding region, is shown in pink. Kink regions are shown in blue in 1J59 and 1OWF; (c) 1RIO - c1 protein-sigma region 4-DNA ternary complex, where distortions at the local level cancel each other out, so that the overall duplex is straight; (d) 1APL - MAT alpha2 homeodomain-DNA complex, where protein-binding does not cause any curvature.
